# Acute effects of pre-exercise high and low glycaemic index meals and exercise timings on substrate metabolism and appetite in postmenopausal women

**DOI:** 10.1038/s41430-025-01615-z

**Published:** 2025-04-15

**Authors:** Miki Sakazaki, Yoshiki Yamada, Yibin Li, Masashi Miyashita

**Affiliations:** 1 Graduate School of Sport Sciences, Waseda University, 2-579-15 Mikajima, Tokorozawa, Saitama 359-1192 Japan; 2 Faculty of Sport Sciences, Waseda University, 2-579-15 Mikajima, Tokorozawa, Saitama 359-1192 Japan; 3 School of Sport, Exercise and Health Sciences, Loughborough University, Loughborough, Leicestershire LE11 3TU UK; 4 Department of Sports Science and Physical Education, The Chinese University of Hong Kong, Shatin, Hong Kong

**Keywords:** Disease prevention, Lifestyle modification

## Abstract

**Background:**

Postmenopausal women are often reported to exhibit increased susceptibility to metabolic disorders. Although the consumption of low glycaemic index (LGI) foods before exercise are known to enhance fat oxidation during exercise, the optimal exercise timing has not been evaluated. We investigated the effects of pre-exercise meals with different GI values and exercise timings on substrate metabolism and appetite in postmenopausal women.

**Methods:**

Fifteen postmenopausal women completed four trials in a random manner. Participants consumed energy-matched high GI (HGI) or LGI meal at 0900 and rested until 1000 (60 min-HGI and -LGI trials) or 1100 (120 min-HGI and -LGI trials). Then, participants performed a 30-min walk at 50% of estimated maximum oxygen uptake and rested until 1300. Expired air, blood samples, ratings of food reward and subjective appetite were collected.

**Results:**

No differences in fat oxidation during exercise were observed among trials (*P* = 0.66). The area under the curve of 3-hydroxybutyrate was higher in the 120 min-LGI trial compared to the 60 min-LGI trial (*P* = 0.01) after exercise. Relative preference for high-fat foods after exercise and subjective appetite during post-exercise for 1-hour were not different between the 60 min-LGI and 120 min-LGI trials.

**Conclusions:**

These findings indicate that a different timing of pre-exercise LGI meal did not influence fat oxidation during exercise, post-exercise food reward or subjective appetite in postmenopausal women. However, a longer interval between meal consumption and subsequent exercise may be effective in enhancing post-exercise hepatic fatty acid oxidation.

## Introduction

An increased risk of dyslipidaemias and metabolic dysfunction-associated steatotic liver disease are recognised as being associated with aging and menopause in women [1, 2]. Fat oxidation rates have been shown to decline in middle-aged and elderly women due to a reduction in lean body mass and reduced skeletal oxidative capacity for lipid metabolism [3, 4]. Therefore, increase in fat utilisation is crucial in preventing metabolic diseases in women.

The consumption of meals with different glycaemic indices (GI) prior to exercise influence subsequence substrate utilisation [5]. Most studies have shown that low GI (LGI) meals enhance fat oxidation and suppress carbohydrate oxidation during exercise compared to high GI (HGI) meals due to a lower postprandial rise in blood glucose and insulin secretion, especially in young men and women with regular exercise habits [5]. In addition, we have previously reported that consuming a mixed meal with LGI 120 min before performing a 60-minute walk enhances fat oxidation in middle-aged women [6]. It is worth noting that the time intervals between the consumption of meal with different GI values and exercise varied widely across studies (ranging from 30 to 180 min) [5]. Although elevated postprandial insulin concentration at the onset of exercise has been known to suppress lipolysis [7], the optimal timing for consuming the LGI meal prior to exercise to maximise fat oxidation during exercise and a post-exercise period compared to the HGI meal has yet to be clarified.

Appetite also plays an important role in maintaining metabolic health. Large fluctuations in blood glucose and insulin in response to a meal increase feelings of hunger or attenuate feelings of satiety [8]. Furthermore, insulin has been reported to suppress food reward-related responses via the modulation of dopamine neuron activity [9]. However, it remains unknown how a LGI meal consumed before exercise influences subjective appetite and food reward in older adults [10].

Therefore, the aim of the present study was to examine the effects of pre-exercise meals with different GI values and exercise timings on substrate oxidation and appetite in postmenopausal women. We hypothesised that the consumption of the LGI meal 60 min relative to 120 min before exercise would enhance fat oxidation during exercise compared to the high GI meal.

## Materials and methods

### Participants

This study was registered in advance with the University Hospital Medical Information Network Center (UMIN), a system for registering clinical trials (ID: UMIN000050447). This study was conducted according to the guidelines laid down in the Declaration of Helsinki. Written informed consent was obtained from all participants. They were Japanese (i.e., self-reported ethnicity) healthy, postmenopausal women. Menopause was defined as 12 months of amenorrhea. None of the participants were having habits of continued “exercise” for at least 30 min/day and at least 2 days/week for at least a year [11]. However, their activities of daily living evaluated by using the Japanese version of International Physical Activity Questionnaire [12] were relatively active through their occupational and household works. The exclusion criteria of the present study were as follows: menstruation, age <40 or >70 years, taking any medication or supplement known to affect substrate metabolism, major illness, current smoker, body mass that had not been stable for at least 3 months before the study or intention to lose weight during the study, participation in other studies while participating in the present study, or history of an immediate allergic reaction to meals (i.e., each food item provided as a test meal in the present study). Participants of the present study were recruited between March 2023 and April 2024 through the distribution of flyers throughout the local community. The descriptive characteristics of the participants (mean ± standard deviation) included in the data analysis were as follows: age 58.0 ± 6.1 years, height 1.59 ± 0.06 m, body mass 59.5 ± 11.5 kg, body mass index 23.7 ± 4.7 kg/m^2^, body fat 33.8 ± 10.2% and estimated maximum oxygen uptake of 30.6 ± 6.7 mL/kg/min.

### Study design and experimental protocol

**Fig. 1:**
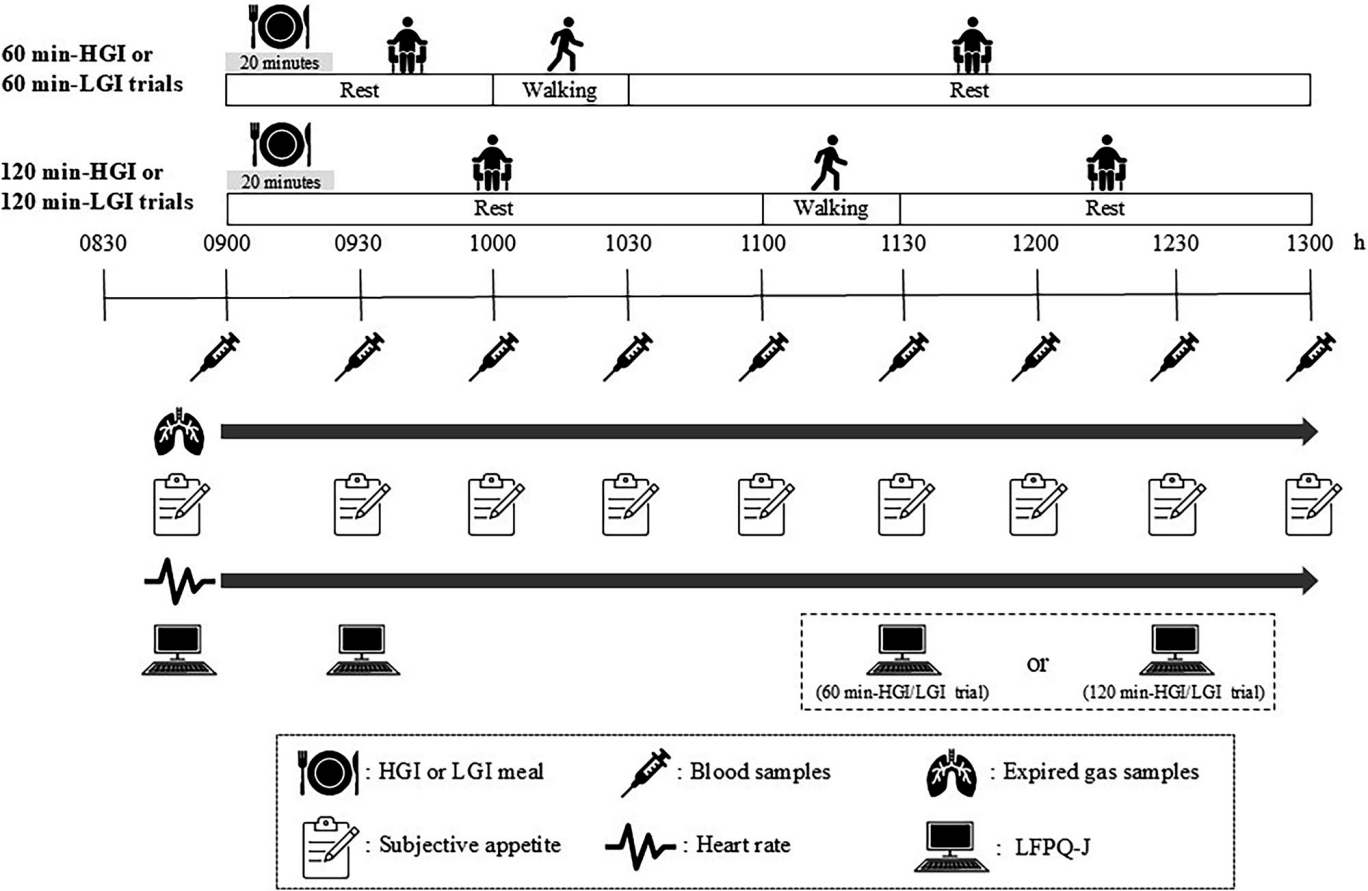
Schematic illustration of the study protocol. HGI high glycaemic index, LGI low glycaemic index, LFPQ-J Leeds Food Preference Questionnaire in Japanese.

Participants completed four, 1-day laboratory-based trials in random order: a 60 min-HGI trial, a 60 min-LGI trial, a 120 min-HGI trial and a 120 min-LGI trial. The lead investigator enrolled the participants in the research and randomly assigned the participants to each trial using computer-generated random numbers. The interval between trials was at least 7 days. A schematic illustration of the study protocol is shown in Fig. 1.

In all trials, all participants reported to the laboratory at 0830 h after a 10-h overnight fast (no food or drink, except water). Body mass was measured to the nearest 0.1 kg using a digital scale (MC-780A-N, Tanita Corporation, Tokyo, Japan). A heart rate monitor (Polar RCX3, Polar Electro, Kempele, Finland) was then fitted to measure heart rate continuously throughout the trial. After a 10-min seated rest, subjective appetite using a paper-based questionnaire (details in “Subjective appetite”), food reward using the Leeds Food Preference Questionnaire in Japanese (LFPQ-J) (details in “Food reward”) and baseline measurements of a 15-min resting expired gas sample were obtained sequentially. Thereafter, a cannula was inserted into the arm vein and a fasting venous blood sample was collected in a seated position. The participants then consumed a HGI or LGI meal at 0900 h. The participants were asked to consume the test meal over 20 min (i.e., the last bite was set at 0920 h), and the order of food intake in each meal was standardised. Subsequently, they were required to rest until 1000 h (60 min-HGI and 60-min-LGI trials) or 1100 h (120 min-HGI and 120 min-LGI trials). During the 30-min walking exercise on the treadmill (JOG NOW 700, Technogym, Cesena, Italy) at a speed eliciting 50% of the estimated maximum oxygen uptake (determined from the preliminary test) (1000–1030 h or 1100–1130 h), oxygen uptake, respiratory exchange ratio (RER), fat oxidation rate and carbohydrate oxidation rate were measured using a stationary gas analyser (Quark RMR, COSMED Co. Ltd., Roma, Italy), and ratings of perceived exertion (RPEs) were assessed periodically [13].

The participants were then asked to sit on a chair in a comfortable position for 90 min or 150 min (1130–1300 h or 1030–1300 h). Further expired gas samples were collected throughout the trial. Also, further venous blood samples were collected and subjective appetite was assessed every 30 min. Furthermore, food rewards using the LFPQ-J were evaluated at 0930 h and 60 min after walking exercise (1130 h or 1230 h) in all trials. The participants consumed water *ad libitum* during the first trial, and the volume ingested was replicated in subsequent trials. Average water intake was 473.4 ± 91.0 mL over the trial.

### Screening and preliminary tests

Body mass and body fat percentage were measured to the nearest 0.1 kg and 0.1%, respectively, using a digital scale (TANITA MC780, Tanita Corporation, Tokyo, Japan). Height was measured using a stadiometre (YS-OA, Yoshida Seisakusho Ltd., Gifu, Japan) to the nearest 0.1 cm. Body mass index was calculated by dividing body mass in kilogrammes by the square of height in metres. Following the anthropometric assessments, the Japanese version of the Three Factor Eating Questionnaire (TFEQ) [14] was conducted as described previously [6]. Thereafter, a screening test of the LFPQ-J, which was developed specifically for the Japanese population, was conducted [15] (details in “Food reward”). The purpose of the screening test was to ask participants about the names of the 16 foods used in the LFPQ-J, their allergies, whether they had ever eaten them and whether they could eat them. After the screening test, participants practiced the tasks performed on the LFPQ-J. Then, resting heart rate was recorded for 1 min after 5 min of seated rest using a short-range telemetry (Polar RCX3, Polar Electro, Kempele, Finland). After familiarisation with the treadmill, each participant was asked to perform a submaximal preliminary exercise test. This test consisted of a 9-min, three-stage incremental walk test to establish the relationship between treadmill speed and oxygen uptake, or heart rate. The initial walking speed was set to 3.0 km/h and was increased by 1.0 or 1.2 km/h every 3 min. The treadmill inclination was set to 0% throughout the test. Oxygen uptake, carbon dioxide production and RER were measured breath-by-breath using a stationary gas analyser (Quark RMR, COSMED, Rome, Italy). Heart rate was measured continuously using a short-range telemetry (Polar RCX3, Polar Electro, Kempele, Finland). RPEs were recorded during the final 15 s of each stage using the Borg scale [13]. Data from the preliminary exercise test were used to estimate the walking workload (i.e., walking speed) corresponding to 50% of each participant’s estimated maximum oxygen uptake, and this workload was used for the main trials.

### Standardisation of dietary intake and physical activity

The participants weighed and recorded all the food and drinks consumed the day before each main trial and refrained from drinking alcohol during this period. They replicated their energy intake from the first to the fourth trial to ensure that it was standardised across trials. Food diaries were analysed using a nutrition analysis software (Excel Eiyoukun Ver 9.0, Kenpakusha, Tokyo, Japan) by a registered dietitian to determine the energy intake and macronutrient content of the foods. Participants were asked to avoid strenuous exercise for one day before each main trial. They wore a uniaxial accelerometer (Lifecoder-EX, Suzuken Co. Ltd., Nagoya, Japan) on their hips to objectively monitor their daily activities during this period as described previously [6].

### Test meals

The contents of the HGI and LGI meals have been described previously [6]. Briefly, the HGI meal consisted of corn flakes containing granulated sugar, skim milk, white bread, margarine, instant mashed potatoes and carbonated drinks (GI value = 73). The LGI meal comprised brown rice with seasoning, skim milk, yogurt, canned peaches, cherry tomatoes and apple juice (GI value = 41). The HGI and LGI meals were adjusted to contain the same energy (33 kJ (7.9 kcal)/kg body mass) and macronutrient content (1.5 g/kg body mass for carbohydrate, 0.1 g/kg body mass for fat and 0.3 g/kg body mass for protein). The energy density of the test meal was 5.4 kJ (1.3 kcal)/g and 2.9 kJ (0.7 kcal)/g for the HGI and LGI meals, respectively.

### Blood analysis

Enzymatic colourimetric assays were used to measure plasma glucose (GLU-HK(M), Shino-Test Corporation, Kanagawa, Japan), serum triglyceride (TG) (Pure Auto S TG-N, Sekisui Medical Co. Ltd., Tokyo, Japan), serum non-esterified fatty acids (NEFA) (NEFA-HR, Wako Pure Chemical Industries Ltd., Osaka, Japan) and serum 3-hydroxybutyrate (KAINOS 3-HB, Kainos Laboratories, Inc., Tokyo, Japan). Enzyme-linked immunosorbent assays (ELISA) were used to measure plasma insulin (Mercodia Insulin ELISA, Mercodia AB, Uppsala, Sweden) and plasma glycerol (Glycerol Colorimetric Assay Kit, Cayman Chemical Company, Michigan, USA). The intra-assay coefficients of variation were 0.8% for glucose, 5.6% for insulin, 1.2% for TG, 0.8% for NEFA, 0.8% for 3-hydroxybutyrate and 4.7% for glycerol.

### Food reward

Food reward was measured using the LFPQ-J, a computer-based task that assesses the different components of food preference and reward [15]. The LFPQ-J measures explicit liking and wanting directly and implicit wanting indirectly, using 16 images of foods that are either high fat savoury, low fat savoury, high fat sweet or low fat sweet. The assessment of food reward using the LFPQ-J was conducted according to procedures described previously [6]. Briefly, the LFPQ-J consists of two tasks: single- and paired-food tasks. Food reward was evaluated using the following four parameters: explicit liking, explicit wanting, implicit wanting and relative preference. For these parameters measured in all food-reward measurements, the fat appeal bias for foods with different fat contents and the taste appeal bias for foods with different tastes were evaluated. Positive values indicate a preference for high fat and/or sweet foods; negative values indicate a preference for low fat and/or savoury foods; and a score of 0 indicates an equal preference between fat content and taste categories [16].

### Subjective appetite

Subjective appetite (satiety, fullness, hunger and prospective food intake) was assessed on a 100-mm visual analogue scale using a paper-based questionnaire (each end of the line represents the most extreme sensation experienced by the participant) [17]. The overall subjective appetite score was calculated from the results of the four appetite ratings using the following equation: Satiety + fullness + (100 - hunger) + (100 - prospective food intake) / 4 [18], where 100 indicates low appetite and 0 indicates high appetite.

### Calculations and statistical analysis

We calculated the required sample size based on data from a previous study [6] using G*Power 3.1.9.7 [19]. The previous study reported a between-trial difference in the total amount of fat oxidation throughout the exercise (2.9 ± 1.9 g/h, mean ± standard deviation) between the HGI trial and the LGI trial in middle-aged women [6]. Based on this study with 15 participants, we calculated that an estimated total sample size of 28 was needed to provide 80% power to detect an effect size of 0.55 (Cohen’s *d*) using a paired *t*-test for comparison between trials, with an alpha level set at 0.05. However, we assumed that a larger effect size (i.e., 0.80) would be attainable for the present study since a greater postprandial glucose and insulin responses might be observed in postmenopausal women as the target population in our study [20]. For two trials with an alpha level set at 0.05 and a correlation of 0.5, an estimated total sample size of 15 would be achieve 80% power to detect between-trial differences. Based on this calculation, 18 participants were recruited to allow for potential withdrawals. Data were analysed using IBM SPSS Statistics for Windows version 29.0.2 (IBM Corp., New York, USA). The rates of fat and carbohydrate oxidation and gross energy expenditure were estimated from oxygen uptake and carbon dioxide production using stoichiometric equations [21]. Total fat and carbohydrate oxidation during the 30-min walking exercise was estimated from the cumulative rate of oxidation for each participant. The total area under the curve (AUC) were calculated using GraphPad Prism version 9.2.0 for Windows (GraphPad Software Inc., California, USA). Generalised estimating equations were used to examine the between-trial differences for all parameters. Where statistically significant differences in baseline values were found, statistical analysis was performed by adjusting for baseline covariates. Where a significant trial-by-time interaction was found, post-hoc pairwise comparisons were performed using the Bonferroni method. The 95% confidence intervals (95% CI) for the mean absolute pairwise differences between trials were calculated using the *t*-distribution and degrees of freedom (*n* - 1). Effect sizes (Cohen’s *d*) were calculated to describe the magnitude of differences between trials ( >0.8, large; 0.5–0.8, moderate; <0.5, small; and <0.2, trivial) [22]. Statistical significance was accepted at the <5% level. Results are reported as mean ± standard deviation.

## Results

### Participants

The participant flow diagram is shown in Supplementary Fig. 1. Eighteen participants signed the informed consent and were randomly assigned to the intervention. One participant dropped out of the study before starting the first main trial because of a schedule conflict and two participants dropped out of the study after the first main trial because of health conditions (not related to this research participation). Fifteen participants completed four trials as described in the methods section and were included in the data analysis. None of the participants reported any discomfort or unusual symptoms.

### Physical activity and energy intake

The accelerometer-recorded physical activity level of the participants for the day prior to the four trials did not differ (total step counts, 120 min-HGI: 7991 ± 4287 vs. 120 min-LGI: 8677 ± 4732 vs. 60 min-HGI: 8417 ± 3718 vs. 60 min-LGI: 7871 ± 3338 steps/day, *P* = 0.08; light physical activity, 120 min-HGI: 67 ± 37 vs. 120 min-LGI: 72 ± 48 vs. 60 min-HGI: 68 ± 37 vs. 60 min-LGI: 64 ± 33 min/day, *P* = 0.01, post-hoc tests of trials did not show differ among the four trials; moderate physical activity, 120 min-HGI: 15 ± 10 vs. 120 min-LGI: 17 ± 11 vs. 60 min-HGI: 17 ± 9 vs. 60 min-LGI: 17 ± 12 min/day, *P* = 0.67; vigorous physical activity, 120 min-HGI: 3 ± 3 vs. 120 min-LGI: 5 ± 7 vs. 60 min-HGI: 5 ± 7 vs. 60 min-LGI: 3 ± 3 min/day, *P* = 0.12). All the participants reported that they had consumed identical foods and drinks on the day before the four trials. The mean self-reported energy intake for the day prior to each trial was 7.1 ± 1.3 MJ/day (57.5 ± 7.9% from carbohydrate, 13.8 ± 3.9% from protein and 28.7 ± 6.9% from fat).

### Oxygen uptake, energy expenditure, heart rate and RPE during walking

There were no differences in oxygen uptake (120 min-HGI: 0.84 ± 0.20 (corresponding to 47.4 ± 7.4% of estimated maximum oxygen uptake) vs. 120 min-LGI: 0.86 ± 0.17 (corresponding to 48.6 ± 7.6% of estimated maximum oxygen uptake) vs. 60 min-HGI: 0.87 ± 0.15 (corresponding to 50.0 ± 6.3% of estimated maximum oxygen uptake) vs. 60 min-LGI: 0.90 ± 0.21 (corresponding to 50.6 ± 6.7% of estimated maximum oxygen uptake) L/min, *P* = 0.60), gross energy expenditure (120 min-HGI: 0.50 ± 0.12 vs. 120 min-LGI: 0.51 ± 0.10 vs. 60 min-HGI:0.52 ± 0.08 vs. 60 min-LGI: 0.54 ± 0.12 MJ, *P* = 0.52), mean heart rate (120 min-HGI: 108 ± 12 vs. 120 min-LGI: 108 ± 13 vs. 60 min-HGI: 113 ± 14 vs. 60 min-LGI: 108 ± 11 beats/min, *P* = 0.36) or RPEs (120 min-HGI: 12 ± 1 vs. 120 min-LGI: 12 ± 1 vs. 60 min-HGI: 12 ± 1 vs. 60 min-LGI: 12 ± 1, *P* = 0.11) during the 30-min walk among trials.

### Substrate oxidation

**Fig. 2:**
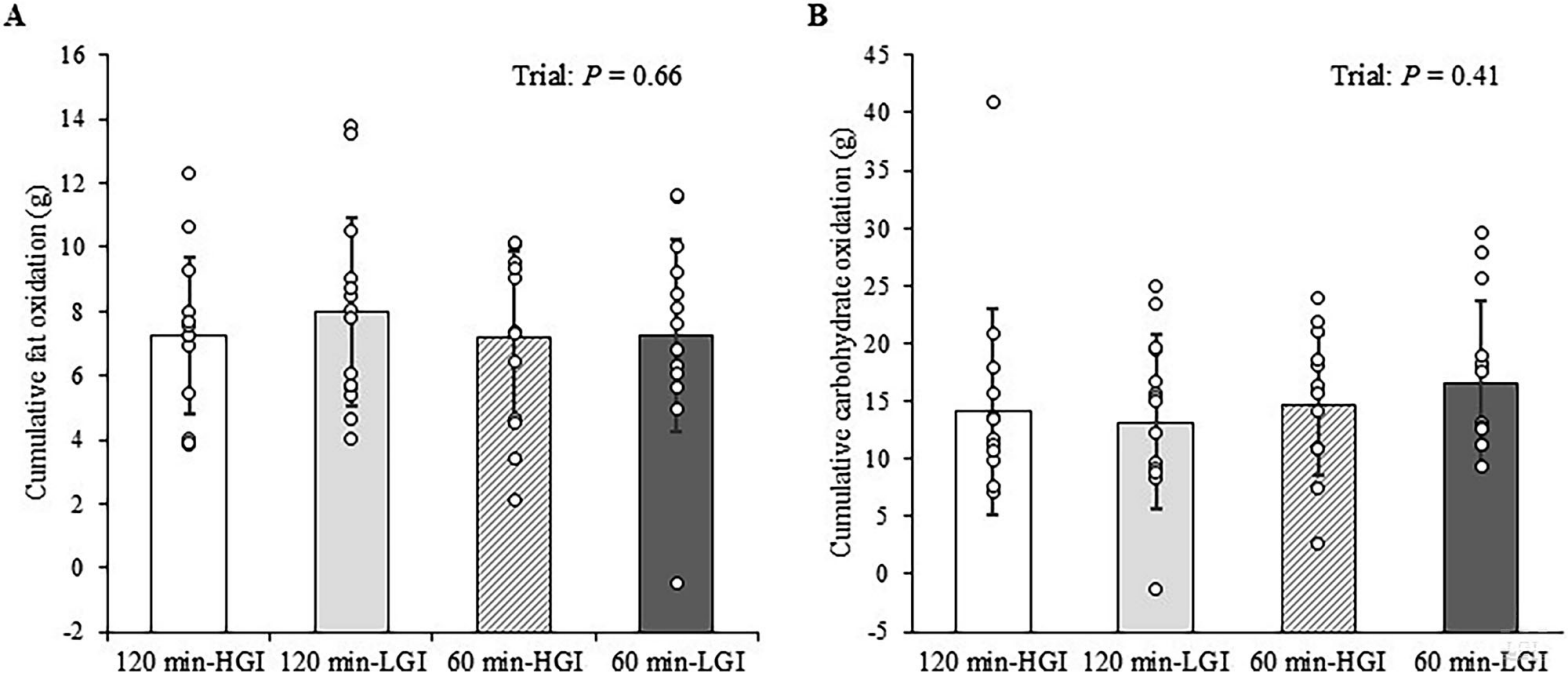
Cumulative substrate oxidation during the 30-min walking exercise in the 120 min-HGI, 120 min-LGI,60 min-HGI and 60 min-LGI trials. **A** Cumulative fat oxidation. **B** Cumulative carbohydrate oxidation. Values are means ± standard deviation. Values were compared using generalised estimating equations. HGI high glycaemic index, LGI low glycaemic index.

The cumulative fat and carbohydrate oxidation values during the 30-min walk (1000–1030 h or 1100–1130 h) are shown in Fig. 2. The cumulative fat and carbohydrate oxidation did not differ among the four trials (*P* = 0.66 and *P* = 0.41, respectively).

The fat and carbohydrate oxidation rates throughout the trial are shown in Supplementary Table 1. The respiratory exchange ratios throughout the trial are shown in Supplementary Table 2.

### Plasma glucose and insulin

**Fig. 3:**
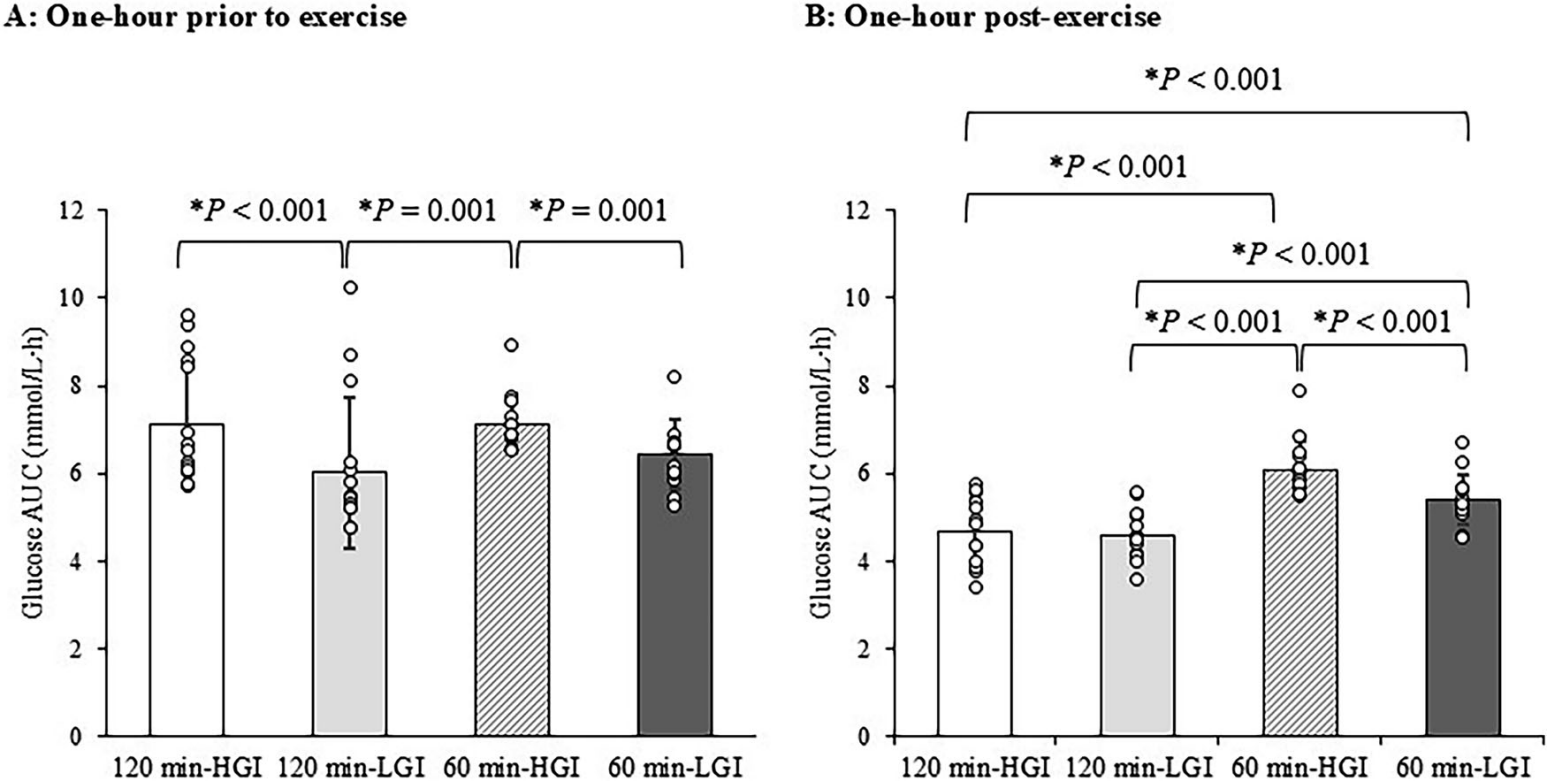
Area under the curve of glucose concentrations in the 120 min-HGI, 120 min-LGI, 60 min-HGI and 60 min-LGI trials. **A** Area under the curve of glucose concentrations measured 1-h prior to exercise periods. **B** Area under the curve of glucose concentrations measured 1-h post-exercise periods. Values are means ± standard deviation. Values were compared using generalised estimating equations. Post hoc analysis was adjusted for multiple comparisons using the Bonferroni method. *Significantly different between trials. HGI high glycaemic index, LGI low glycaemic index, AUC area under the curve.

The circulating concentrations of plasma glucose in the 120 min-HGI and 120-min LGI trials (A), and the 60 min-HGI and 60 min-LGI trials (B) are shown in Supplementary Fig. 2. The AUC of glucose concentrations during pre-exercise for 1-hour (A) and post-exercise for 1-hour (B) in the four trials are shown in Fig. 3. For Fig. 3B, the AUC of glucose concentrations during post-exercise for 1-hour were lower in the 120 min-HGI trial than in the 60 min-HGI trial (95% CI −1.9 to −0.9 mmol/L⋅h, *P* < 0.001, ES = 0.13), and in the 120 min-LGI trial than in the 60 min-LGI trial (95% CI −1.2 to −0.3 mmol/L⋅h, *P* < 0.001, ES = 1.29).

**Fig. 4:**
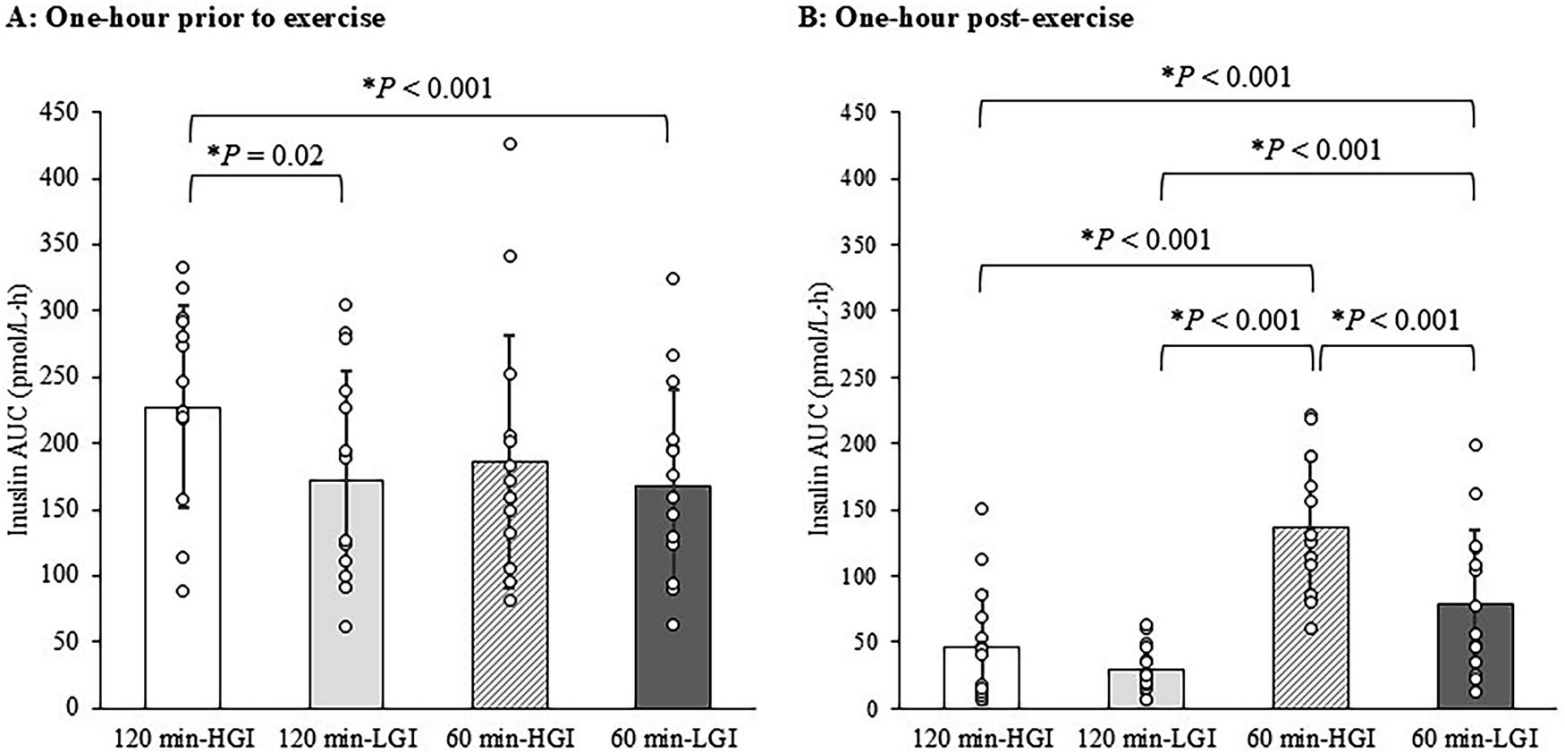
Area under the curve of insulin concentrations in the 120 min-HGI, 120 min-LGI, 60 min-HGI and 60 min-LGI trials. **A** Area under the curve of insulin concentrations measured 1-h prior to exercise periods. **B** Area under the curve of insulin concentrations measured 1-h post-exercise periods. Values are means ± standard deviation. Values were compared using generalised estimating equations. Post hoc analysis was adjusted for multiple comparisons using the Bonferroni method. *Significantly different between trials. HGI high glycaemic index, LGI low glycaemic index, AUC area under the curve.

The circulating concentrations of plasma insulin in the 120 min-HGI and 120-min LGI trials (A), and the 60 min-HGI and 60 min-LGI trials (B) are shown in Supplementary Fig. 3. The AUC of insulin concentrations during pre-exercise for 1-hour (A) and post-exercise for 1-hour (B) in the four trials are shown in Fig. 4. For Fig. 4B, the AUC of insulin concentrations during post-exercise for 1-hour were lower in the 120 min-HGI trial than in the 60 min-HGI trial (95% CI −123.3 to −56.8 pmol/L⋅h, *P* < 0.001, ES = 0.56), and in the 120 min-LGI trial than in the 60 min-LGI trial (95% CI −76.4 to −21.5 pmol/L⋅h, *P* < 0.001, ES = 1.25).

### Serum TG, NEFA and 3-hydroxybutyrate, and plasma glycerol

**Fig. 5:**
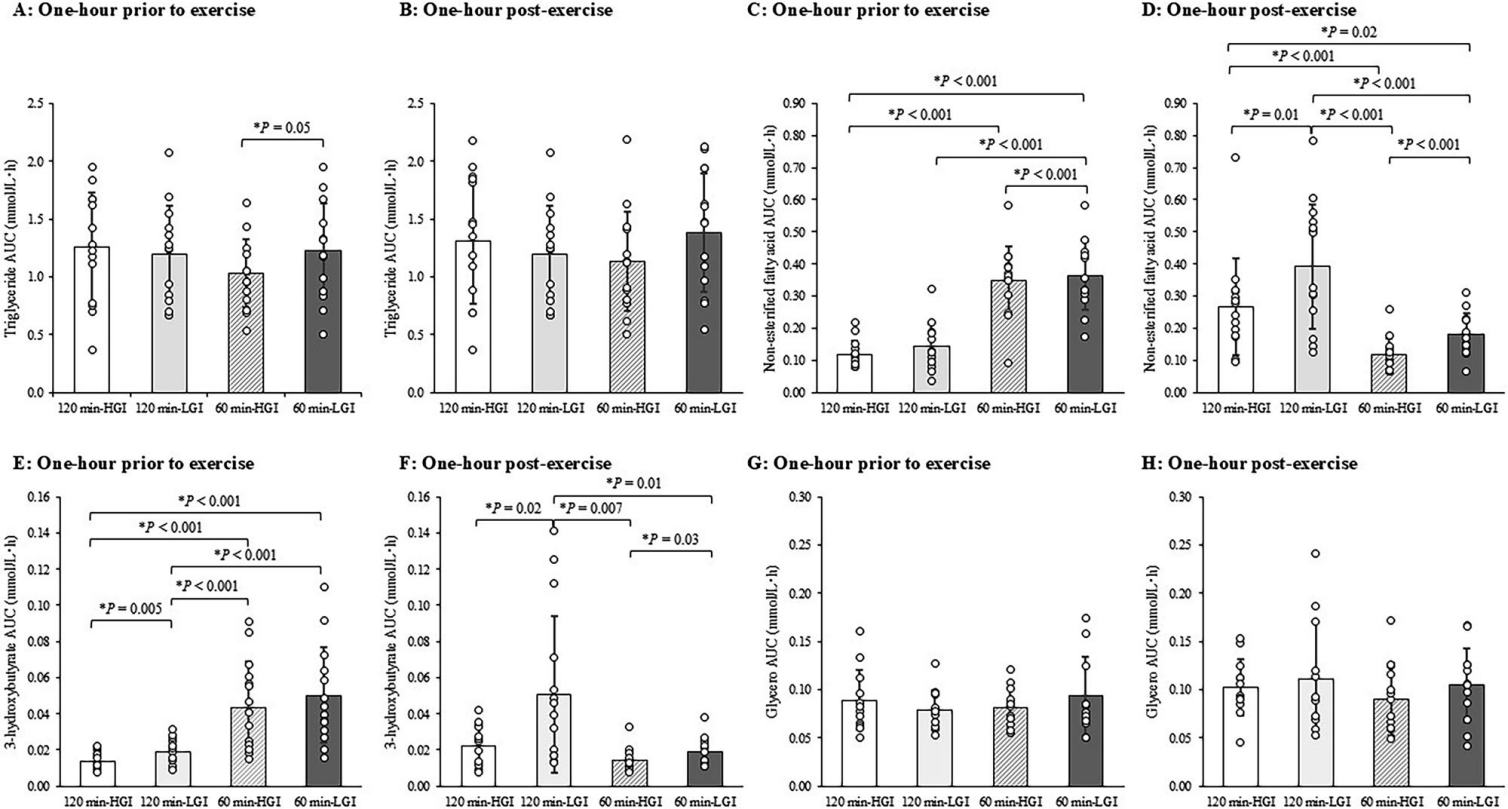
Area under the curve of triglyceride, non-esterified fatty-acid, 3-hydroxybutyrate and glycerol concentrations in the 120 min-HGI, 120 min-LGI, 60 min-HGI and 60 min-LGI trials. **A**, **C**, **E**, **G** Area under the curve of triglyceride, non-esterified fatty-acid, 3-hydroxybutyrate and glycerol concentrations measured 1-h prior to exercise periods. **B**, **D**, **F**, **H** Area under the curve of triglyceride, non-esterified fatty-acid, 3-hydroxybutyrate and glycerol concentrations measured 1-h post-exercise periods. Values are means ± standard deviation. Values were compared using generalised estimating equations. Post hoc analysis was adjusted for multiple comparisons using the Bonferroni method. *Significantly different between trials. HGI high glycaemic index, LGI low glycaemic index, AUC area under the curve.

The circulating concentrations of serum TG in the 120 min-HGI and 120-min LGI trials, and the 60 min-HGI and 60 min-LGI trials are shown in Supplementary Fig. 4. The AUC of TG concentrations during pre-exercise for 1-hour (A) and post-exercise for 1-hour (B) in the four trials are shown in Fig. 5. For Fig. 5B, a significant main effect of trial was observed for the AUC of TG concentrations during post-exercise for 1-hour (*P* = 0.006). Subsequent post-hoc tests did not reveal between-trial differences.

The circulating concentrations of serum NEFA in the 120 min-HGI and 120-min LGI trials, and the 60 min-HGI and 60 min-LGI trials are shown in Supplementary Fig. 5. The AUC of NEFA concentrations during pre-exercise for 1-hour (C) and post-exercise for 1-hour (D) in the four trials are shown in Fig. 5. For Fig. 5D, the AUC of NEFA concentrations during post-exercise for 1-hour were higher in the 120 min-HGI trial than in the 60 min-HGI trial (95% CI 0.05 to 0.3 mmol/L⋅h, *P* < 0.001, ES = 0.78), and in the 120 min-LGI trial than in the 60 min-LGI trial (95% CI 0.09 to 0.3 mmol/L⋅h, *P* < 0.001, ES = 1.08).

The circulating concentrations of serum 3-hydroxybutyrate in the 120 min-HGI and 120-min LGI trials, and the 60 min-HGI and 60 min-LGI trials are shown in Supplementary Fig. 6. The AUC of 3-hydroxybutyrate concentrations during pre-exercise for 1-hour (E) and post-exercise for 1-hour (F) in the four trials are shown in Fig. 5. For Fig. 5F, the AUC of 3-hydroxybutyrate concentrations during post-exercise for 1-hour was higher in the 120 min-LGI trial than in the 60 min-LGI trial (95% CI 0.004 to 0.06 mmol/L⋅h, *P* = 0.01, ES = 0.81).

The circulating concentrations of plasma glycerol in the 120 min-HGI and 120-min LGI trials, and the 60 min-HGI and 60 min-LGI trials are shown in Supplementary Fig. 7. The AUC of glycerol concentrations during pre-exercise for 1-hour (G) and post-exercise for 1-hour (H) in the four trials are shown in Fig. 5. For Fig. 5H, the AUC of glycerol concentrations during post-exercise for 1-hour did not differ among the four trials (*P* = 0.09).

### Food reward

Explicit liking, explicit wanting, implicit wanting and relative preference for fat appeal and taste appeal biases in each trial are shown in Table 1. Post-hoc tests of an interaction effect revealed that explicit liking for fat appeal bias were lower in the 120 min-HGI trial than in the 120 min-LGI trial and 60 min-HGI trial at 0930 (95% CI −15.0 to −6.2, *P* < 0.001, ES = 1.58; 95% CI −11.8 to −0.02, *P* = 0.05, ES = 0.66). Post-hoc tests of an interaction effect revealed that explicit wanting for fat appeal bias were lower in the 120 min-HGI trial than in the 120 min-LGI trial at 0930 (95% CI −11.8 to −1.5, *P* = 0.004, ES = 0.85). Post-hoc tests of an interaction effect revealed that relative preference for fat appeal bias were higher in the 120 min-LGI trial than in the 120 min-HGI trial and 60 min-HGI trial at 1-hour after exercise (95% CI 0.6 to 9.8, *P* = 0.02, ES = 0.74; 95% CI 0.2 to 14.5, *P* = 0.04, ES = 0.67). No differences among the four trials were found in explicit liking, explicit wanting, implicit wanting or relative preference for taste appeal bias.

**Table 1:** Explicit liking, explicit wanting, implicit wanting, and relative preference of fat appeal bias and taste appeal bias in the 120 min-HGI, 120 min-LGI, 60 min-HGI and 60 min-LGI trials.

Time (hours)	0900				0930				1-hour after exercise (1130 or 1230)			
	120 min-HGI	120 min-LGI	60 min-HGI	60 min-LGI	120 min-HGI	120 min-LGI	60 min-HGI	60 min-LGI	120 min-HGI	120 min-LGI	60 min-HGI	60 min-LGI
**Fat appeal bias**												
Explicit liking#	−7.4 ± 14.3	−1.1 ± 11.9	−0.1 ± 13.6	−3.5 ± 14.5	−5.8 ± 10.0a	4.8 ± 12.0b	0.2 ± 10.4b	−2.0 ± 10.3	−0.5 ± 12.7	3.8 ± 14.7	1.4 ± 11.7	2.5 ± 8.9
Explicit wanting	−5.4 ± 12.9	−2.6 ± 13.6	−1.7 ± 15.2	−6.3 ± 18.1	−2.5 ± 9.1a	4.2 ± 12.0b	−1.2 ± 12.6	0.3 ± 10.2	1.8 ± 11.2	4.8 ± 13.1	0.9 ± 12.3	0.8 ± 13.1
Implicit wanting	−13.4 ± 31.4	−4.9 ± 34.3	−5.2 ± 35.5	−8.9 ± 37.8	−19.1 ± 23.7	−1.8 ± 29.0	−11.9 ± 35.6	−13.7 ± 27.1	−2.1 ± 28.0	9.5 ± 34.5	−8.5 ± 26.2	−3.0 ± 30.8
Relative preference#	−6.1 ± 13.8	−1.5 ± 13.8	−1.9 ± 15.8	−4.0 ± 16.4	−7.3 ± 9.6	−1.3 ± 12.9	−4.3 ± 15.5	−5.7 ± 11.4	−1.1 ± 12.0a	4.1 ± 14.1b	−3.2 ± 12.5a	−1.7 ± 13.3
**Taste appeal bias**												
Explicit liking	−3.1 ± 15.0	1.4 ± 15.2	−0.2 ± 11.4	0.2 ± 6.8	6.1 ± 8.0	7.6 ± 9.4	4.4 ± 5.7	9.1 ± 13.3	5.7 ± 14.9	1.0 ± 15.2	7.0 ± 11.4	4.7 ± 13.4
Explicit wanting	−1.7 ± 16.3	7.7 ± 9.2	−1.5 ± 12.5	0.0 ± 10.3	7.7 ± 9.2	7.1 ± 11.4	5.1 ± 7.6	11.4 ± 14.7	2.1 ± 16.1	−0.2 ± 15.3	7.0 ± 12.0	5.5 ± 14.9
Implicit wanting	−6.7 ± 27.8	25.4 ± 34.2	−13.1 ± 30.1	−8.5 ± 27.4	25.4 ± 34.2	22.4 ± 35.6	11.8 ± 29.4	22.0 ± 29.8	12.9 ± 39.7	−4.0 ± 32.4	14.1 ± 26.3	7.8 ± 38.9
Relative preference	−3.3 ± 11.6	11.2 ± 12.4	−5.3 ± 11.7	−3.5 ± 11.1	11.1 ± 12.4	7.5 ± 12.6	4.9 ± 11.8	9.7 ± 11.7	2.6 ± 15.9	1.0 ± 14.7	4.9 ± 10.9	3.2 ± 15.8

Values are means ± standard deviation. Values were compared using generalised estimating equations and post-hoc analysis was adjusted for multiple comparisons using the Bonferroni method. #Significantly different among trials, a main effect of trial, *P* ≤ 0.03. Explicit liking and relative preference for fat appeal bias were higher in the 120 min-LGI trial compared to the 120 min-HGI trial (*P* ≤ 0.001 and *P* = 0.04, respectively). Different lowercase letters in the same row at the same time point indicate significant differences between trials (*P* ≤ 0.05).

*HGI* high glycaemic index, *LGI* low glycaemic index.

### Subjective appetite

**Fig. 6:**
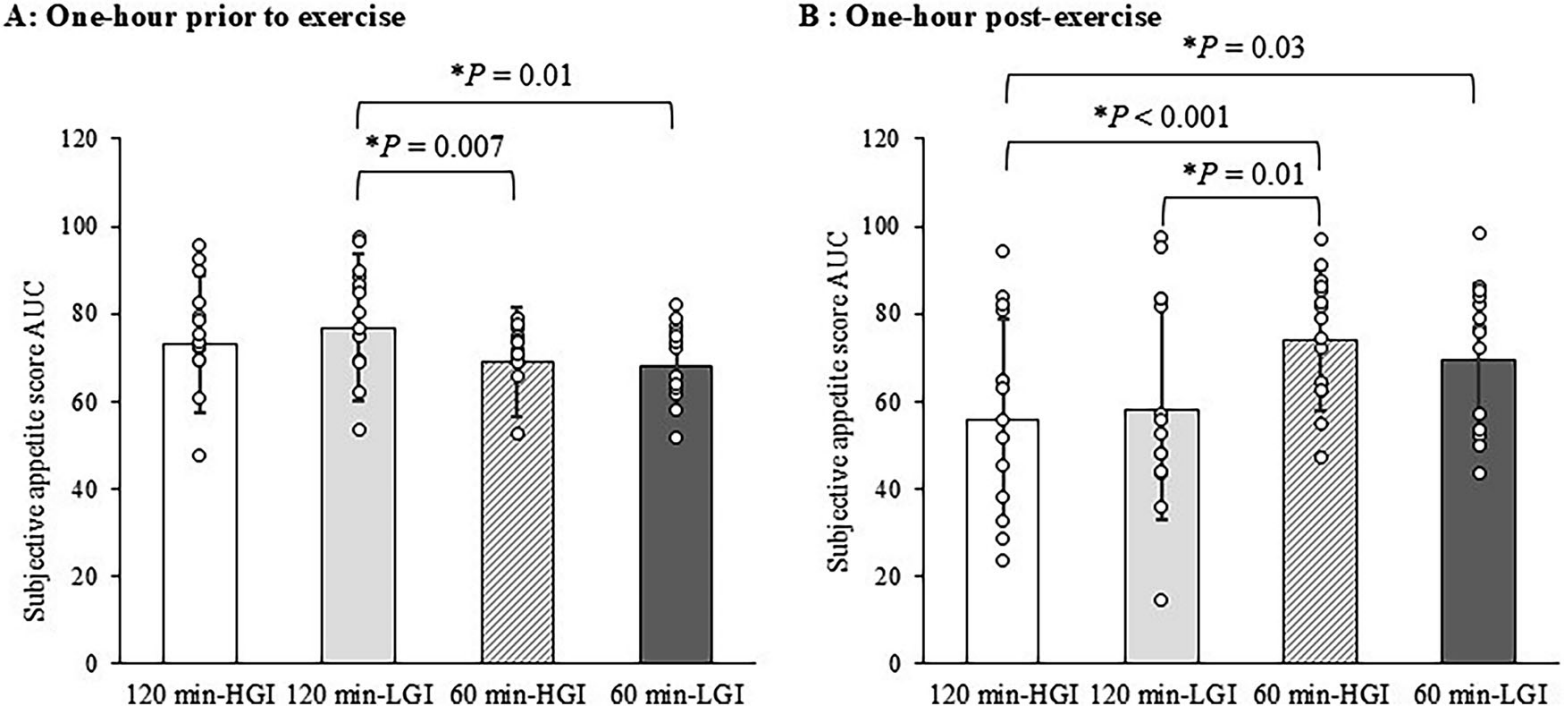
Area under the curve of subjective appetite score in the 120 min-HGI, 120 min-LGI, 60 min-HGI and 60 min-LGI trials. **A** Area under the curve of subjective appetite score measured 1-h prior to exercise periods. **B** Area under the curve of subjective appetite score measured 1-h post-exercise periods. Values are means ± standard deviation. Values were compared using generalised estimating equations. Post hoc analysis was adjusted for multiple comparisons using the Bonferroni method. *Significantly different between trials. HGI high glycaemic index, LGI low glycaemic index, AUC area under the curve.

The AUC of subjective appetite score during pre-exercise for 1-hour (A) and post-exercise for 1-hour (B) in the four trials are shown in Fig. 6. For Fig. 6A, the AUC of subjective appetite score during pre-exercise for 1-hour was higher in the 120 min-LGI trial than in the 60 min-LGI trial (95% CI 1.4 to 16.6, *P* = 0.01, ES = 0.81). For Fig. 6B, the AUC of subjective appetite score during post-exercise for 1-hour was lower in the 120 min-HGI trial than in the 60 min-HGI trial (95% CI −29.6 to −6.9, *P* < 0.001, ES = 1.06). The subjective appetite scores for each trial are listed in Supplementary Table 3.

## Discussion

The primary finding of the present study revealed that a different timing of pre-exercise LGI meal did not affect fat oxidation during a subsequent 30-min walk in postmenopausal women. In addition, elevated hepatic fat oxidation determined by circulating concentrations of 3-hydroxybutyrate and lowered insulin concentration were observed in the 120 min-LGI trial compared to the 60 min-LGI trial after walking in the present study. Furthermore, relative preference for high-fat foods and subjective appetite were not different in response to different timings of LGI when these outcomes were evaluated after walking for a 1-hour period. These findings underscore that although a different timing of pre-exercise LGI meal may not influence substrate oxidation during exercise and subsequent food reward, a longer interval between meal consumption and subsequent exercise may be effective in enhancing post-exercise hepatic fatty acid oxidation, due in part to lowered insulin.

Contrary to our hypothesis, no differences in fat oxidation during exercise were observed among the four trials. This finding may be explained by several reasons. First, the differences in postprandial insulin concentrations were likely insufficient to affect fat and carbohydrate oxidation during walking in the present study. Indeed, no differences were observed in the AUC of insulin concentrations during the pre-exercise period between trials with different exercise timings (i.e., 120 min vs. 60 min). A previous study has demonstrated that homeostasis model assessment of insulin resistance increases in postmenopausal women [23]. This finding suggests that elevated secretion of insulin may occur in postmenopausal women and we indeed observed that peak insulin concentrations following the consumption of a LGI meal in the present study were elevated compared to those observed in the previous study conducted with the identical meal in middle-aged women (241.4 ± 97.0 pmol/L in 120 min-LGI and 225.8 ± 104.5 pmol/L in 60 min-LGI vs. 193.8 ± 88.2 pmol/L in the previous study [6]). Therefore, increased pre-exercise insulin concentrations may have suppressed fat oxidation during exercise [7], even following LGI meal consumption, in postmenopausal women. Another potential reason is that the exercise duration in this study may not have been designed long enough to demonstrate cumulative differences in substrate oxidation. In the present study, the intensity of exercise was designed to elicit maximal fat oxidation [24]. Previous studies, which conducted a 60-min exercise session at low- to moderate-intensity, have reported higher cumulative fat oxidation after LGI meals compared to HGI meals in young adults [25, 26]. Nevertheless, extending the duration or increasing the intensity of exercise is challenging for older adults. Collectively, our findings provide critical insights and future avenues for the prevention of age-associated insulin resistance to enhance the efficacy of dietary interventions along with exercise in older women.

Conversely, in the post-exercise period, the AUC of NEFA and 3-hydroxybutyrate concentrations were observed to be higher in the 120 min-HGI trial compared to the 60 min-HGI trial, as well as in the 120 min-LGI trial compared to the 60 min-LGI trial in the present study. These findings suggest that extending the interval between meal consumption and exercise may enhance post-exercise fat metabolism [27]. Since an increase in the AUC of insulin concentrations during the post-exercise period was observed in the 60 min-HGI trial and the 60 min-LGI trial, elevated insulin concertation following exercise may have inhibited lipolysis [28]. This response is likely due to variations in the rate of carbohydrate digestion and absorption in the small intestine, as well as fluctuations of glucose concentrations [29] among trials following different timings of exercise. Consequently, consuming a LGI meal 120 min relative to 60 min prior to exercise may be more effective in promoting hepatic fat oxidation determined by circulating concentrations of 3-hydroxybutyrate during the post-exercise period.

We observed that explicit liking and wanting for fat appeal bias scores were lower (i.e., reward bias away from high-fat towards low-fat foods) in the 120 min-HGI trial compared to the 120 min-LGI trial after the meal. While the precise mechanisms by which insulin influences food reward within the central nervous system remain to be fully elucidated, one plausible mechanism is that insulin enhances synaptic activity of the mesolimbic dopamine reuptake transporter, suggesting elevated insulin secretion diminishes the rewarding aspect of food by decreasing dopamine signaling [30]. Thus, we assume that an increase in insulin concentration following the consumption of a HGI meal could suppress hedonic appeal for high-fat foods. With respect to the timing of exercise, elevated relative preference for fat appeal bias score during the post-exercise period was observed in the 120 min-HGI trial compared to the 120 min-LGI trial, but not between the 60 min-HGI trial and the 60 min-LGI trial whilst there was a difference in insulin concentration. In terms of the effects of exercise on food reward, previous studies have demonstrated that acute moderate-intensity (i.e., 70% of maximum oxygen uptake or 68% of peak oxygen uptake) running decreases the preference for high-fat and sweet foods [31] and enhances reactivity to low-density foods in certain brain regions associated with the reward system [32]. Although these previous studies have been performed in a different exercise intensity and energy state (i.e., higher-intensity exercise and a fasting state), exercise on its own may lower relative preference for fat appeal bias in the present study. Therefore, exercise performed 60 min after a meal may be effective in suppressing the preference for high-fat foods in both the HGI and LGI trials regardless the presence of insulin. However, it is worth noting that despite the consumption of an identical meal, a difference in explicit liking for fat appeal bias score was observed between the 120 min-HGI trial and the 60 min-HGI trial measured immediately after a meal, likely due to substantial interindividual differences. Furthermore, no significant differences were noted in taste appeal bias score among trials. Since our study design cannot allow us to distinguish the effects of GI meal and exercise on food reward, additional studies are required to elucidate our speculations as discussed. On the other hand, the AUC of subjective appetite score was observed to be lower in the 120 min-HGI trial compared to the 60 min-HGI trial after exercise. This finding indicates that exercise performed 60 min post-meal, as compared to 120 min, leads to a transient suppression of subjective appetite due to elevated insulin concentrations observed following exercise which is associated with reduced hunger and increased fullness [33]. Collectively, our findings imply that a shorter interval between meal consumption and subsequent exercise may be more advantageous for the suppression of both hedonic appetite (i.e., high-fat foods relative to low-fat foods) and homeostatic appetite (i.e., subjective appetite).

A strength of the present study is that we were the first to investigate the optimal timing of exercise following the consumption of meals with different GI values, specifically aimed at enhancing fat metabolism during exercise. Furthermore, our study focused on postmenopausal women, a population with an elevated risk of metabolic diseases [34], in contrast to most previous studies that have targeted young men and women [5]. Despite these strengths, it is important to note that the estimated GI values of the mixed meals may not have been truly reflected the postprandial glucose and insulin responses in this population, partly due to estrogen-related progression in impaired glucose metabolism [23, 35] with considerable inter-individual variation. In addition, the absence of a control condition without exercise (i.e., total resting condition) makes it difficult to distinguish the effects between the meal and exercise. Future research should investigate the effects of different GI meals and exercise timings in other populations, specifically older adults including men, to suggest the dietary and exercise recommendations for maintaining metabolic health.

In conclusion, the present study demonstrates that a different timing of LGI meal did not influence fat oxidation during a 30-min walking exercise, post-exercise food reward or subjective appetite in postmenopausal women. However, the consumption of a LGI meal 120 min prior to exercise, as compared to 60 min, enhances hepatic fatty acid oxidation in the post-exercise period.

## Supplementary information

Supplemental Figures and Tables_revised

## Data Availability

All data sets generated during and/or analysed during the present study are not publicly available but are available from the corresponding author on reasonable request.

## References

[1] Goh VH, Tong TY, Mok HP, Said B. Differential impact of aging and gender on lipid and lipoprotein profiles in a cohort of healthy Chinese Singaporeans. Asian J Androl. 2007;9:787–94. 17968464 10.1111/j.1745-7262.2007.00294.x

[2] Cao W, Xu Y, Shen Y, Wang Y, Ma X, Bao Y. Associations between sex hormones and metabolic-associated fatty liver disease in a middle-aged and elderly community. Endocr J. 2022;69:1007–14. 35321990 10.1507/endocrj.EJ21-0559

[3] Calles-Escandón J, Arciero PJ, Gardner AW, Bauman C, Poehlman ET. Basal fat oxidation decreases with aging in women. J Appl Physiol. 1985;78:266–71. 10.1152/jappl.1995.78.1.266 7713822

[4] Levadoux E, Morio B, Montaurier C, Puissant V, Boirie Y, Fellmann N, et al. Reduced whole-body fat oxidation in women and in the elderly. Int J Obes Relat Metab Disord. 2001;25:39–44. 11244456 10.1038/sj.ijo.0801530

[5] Burdon CA, Spronk I, Cheng HL, O’Connor HT. Effect of glycemic index of a pre-exercise meal on endurance exercise performance: a systematic review and meta-analysis. Sports Med. 2017;47:1087–101. 27677914 10.1007/s40279-016-0632-8

[6] Sakazaki M, Yoshikawa Y, Kamemoto K, Tataka Y, Yamada Y, Wu CL, et al. Effects of pre-exercise high and low glycaemic index meals on substrate metabolism and appetite in middle-aged women. J Nutr Sci. 2023;12:e114. 38025305 10.1017/jns.2023.96 PMC10660074

[7] Horowitz JF, Mora-Rodriguez R, Byerley LO, Coyle EF. Lipolytic suppression following carbohydrate ingestion limits fat oxidation during exercise. Am J Physiol. 1997;273:E768–75. 9357807 10.1152/ajpendo.1997.273.4.E768

[8] Niwano Y, Adachi T, Kashimura J, Sakata T, Sasaki H, Sekine K, et al. Is glycemic index of food a feasible predictor of appetite, hunger, and satiety?. J Nutr Sci Vitaminol. 2009;55:201–7. 19602827 10.3177/jnsv.55.201

[9] Edwin Thanarajah S, Iglesias S, Kuzmanovic B, Rigoux L, Stephan KE, Brüning JC, et al. Modulation of midbrain neurocircuitry by intranasal insulin. Neuroimage. 2019;194:120–7. 30914385 10.1016/j.neuroimage.2019.03.050

[10] Bennett C, Burrows T, Pursey K, Poudel G, Ng KW, Nguo K, et al. Neural responses to food cues in middle to older aged adults: a scoping review of fMRI studies. Nutr Diet. 2021;78:343–64. 33191542 10.1111/1747-0080.12644

[11] Ministry of Health, Labour and Welfare, Japan [Internet]. The National Health and Nutrition Survey in Japan. Tokyo, Japan: Ministry of Health, Labour and Welfare; 2019. [accessed 15 October 2024].

[12] Murase N, Katsumura T, Ueda C, Inoue S, Shimomitsu T. Validity and reliability of Japanese version of International Physical Activity Questionnaire. J Health Welf. 2002;49:1–9.

[13] Borg GA. Perceived exertion: a note on “history” and method. Med Sci Sports. 1973;5:90–3. 4721012

[14] Adachi Y, Fujii K, Yamagami T. Responses regarding restrained eating on the Three-Factor Eating Questionnaire and weight loss. Jap J Behav Ther. 1992;18:54–66.

[15] Hiratsu A, Thivel D, Beaulieu K, Finlayson G, Nagayama C, Kamemoto K, et al. Development of the Leeds Food Preference Questionnaire in Japanese: sensitivity and reproducibility of liking and wanting for food in fasted and fed states. Food Qual Prefer. 2022;102:104677.

[16] Oustric P, Thicel D, Dalton M, Beaulieu K, Gibbons C, Hopkins M, et al. Measuring food preference and reward: application and cross-cultural adaptation of the Leeds Food Preference Questionnaire in human experimental research. Food Qual Prefer. 2020;80:103824.

[17] Flint A, Raben A, Blundell JE, Astrup A. Reproducibility, power and validity of visual analogue scales in assessment of appetite sensations in single test meal studies. Int J Obes Relat Metab Disord. 2000;24:38–48. 10702749 10.1038/sj.ijo.0801083

[18] Gibbons C, Hopkins M, Beaulieu K, Oustric P, Blundell JE. Issues in measuring and interpreting human appetite (Satiety/Satiation) and its contribution to obesity. Curr Obes Rep. 2019;8:77–87. 31037612 10.1007/s13679-019-00340-6 PMC6517339

[19] Faul F, Erdfelder E, Lang AG, Buchner A. G*Power 3: a flexible statistical power analysis program for the social, behavioral, and biomedical sciences. Behav Res Methods. 2007;39:175–91. 17695343 10.3758/bf03193146

[20] Bermingham KM, Linenberg I, Hall WL, Kadé K, Franks PW, Davies R, et al. Menopause is associated with postprandial metabolism, metabolic health and lifestyle: The ZOE PREDICT study. eBioMedicine. 2022;85:104303. 36270905 10.1016/j.ebiom.2022.104303 PMC9669773

[21] Frayn KN. Calculation of substrate oxidation rates in vivo from gaseous exchange. J Appl Physiol Respir Environ Exerc Physiol. 1983;55:628–34. 6618956 10.1152/jappl.1983.55.2.628

[22] Cohen J Statistical power analysis for the behavioral sciences. Second ed. Hillsdale NJ: Lawrence Erlbaum Associates; 1988.

[23] Oya J, Nakagami T, Yamamoto Y, Fukushima S, Takeda M, Endo Y, et al. Effects of age on insulin resistance and secretion in subjects without diabetes. Intern Med. 2014;53:941–7. 24785884 10.2169/internalmedicine.53.1580

[24] Stisen AB, Stougaard O, Langfort J, Helge JW, Sahlin K, Madsen K. Maximal fat oxidation rates in endurance trained and untrained women. Eur J Appl Physiol. 2006;98:497–506. 17006714 10.1007/s00421-006-0290-x

[25] Stevenson EJ, Astbury NM, Simpson EJ, Taylor MA, Macdonald IA. Fat oxidation during exercise and satiety during recovery are increased following a low-glycemic index breakfast in sedentary women. J Nutr. 2009;139:890–7. 19321590 10.3945/jn.108.101956

[26] Sun FH, Wong SHS, Huang YJ, Chen YJ, Tsang KF. Substrate utilization during brisk walking is affected by glycemic index and fructose content of a pre-exercise meal. Eur J Appl Physiol. 2012;112:2565–74. 22081046 10.1007/s00421-011-2231-6

[27] Karpe F, Dickmann JR, Frayn KN. Fatty acids, obesity, and insulin resistance: time for a reevaluation. Diabetes. 2011;60:2441–9. 21948998 10.2337/db11-0425 PMC3178283

[28] Dimitriadis G, Mitrou P, Lambadiari V, Maratou E, Raptis SA. Insulin effects in muscle and adipose tissue. Diabetes Res Clin Pr. 2011;93:S52–9. 10.1016/S0168-8227(11)70014-6 21864752

[29] Chacko E. Exercising tactically for taming postmeal glucose surges. Scientifica. 2016;2016:4045717. 27073714 10.1155/2016/4045717 PMC4814694

[30] Figlewicz DP, Benoit SC. Insulin, leptin, and food reward: update 2008. Am J Physiol Regul Integr Comp Physiol. 2009;296:R9–19. 10.1152/ajpregu.90725.2008 PMC2636975 18945945

[31] Yamada Y, Hiratsu A, Thivel D, Beaulieu K, Finlayson G, Nagayama C, et al. Reward for fat and sweet dimensions of food are altered by an acute bout of running in healthy young men. Appetite. 2024;200:107562. 38880282 10.1016/j.appet.2024.107562

[32] Thackray AE, Hinton EC, Alanazi TM, Dera AM, Fujihara K, Hamilton-Shield JP, et al. Exploring the acute effects of running on cerebral blood flow and food cue reactivity in healthy young men using functional magnetic resonance imaging. Hum Brain Mapp. 2023;44:3815–32. 37145965 10.1002/hbm.26314 PMC10203797

[33] Flint A, Gregersen NT, Gluud LL, Møller BK, Raben A, Tetens I, et al. Associations between postprandial insulin and blood glucose responses, appetite sensations and energy intake in normal weight and overweight individuals: a meta-analysis of test meal studies. Br J Nutr. 2007;98:17–25. 17524176 10.1017/S000711450768297X

[34] Marsh ML, Oliveira MN, Vieira-Potter VJ. Adipocyte metabolism and health after the menopause: The role of exercise. Nutrients. 2023;15:444. 36678314 10.3390/nu15020444 PMC9862030

[35] Kawakami M, Yokota-Nakagi N, Uji M, Yoshida K, Tazumi S, Takamata A, et al. Estrogen replacement enhances insulin-induced AS160 activation and improves insulin sensitivity in ovariectomized rats. Am J Physiol Endocrinol Metab. 2018;315:E1296–304. 10.1152/ajpendo.00131.2018 30179516

