# Supplementary material for: Acute effects of pre-exercise high and low glycaemic index meals and exercise timings on substrate metabolism and appetite in postmenopausal women
Source: Eur J Clin Nutr. 2025 Apr 15;79(12):1218–26. doi: 10.1038/s41430-025-01615-z (PMC12678173; doi:10.1038/s41430-025-01615-z)
Supplement: Supplementary file 1 — Supplemental Figures and Tables_revised [file 41430_2025_1615_MOESM1_ESM.pdf]

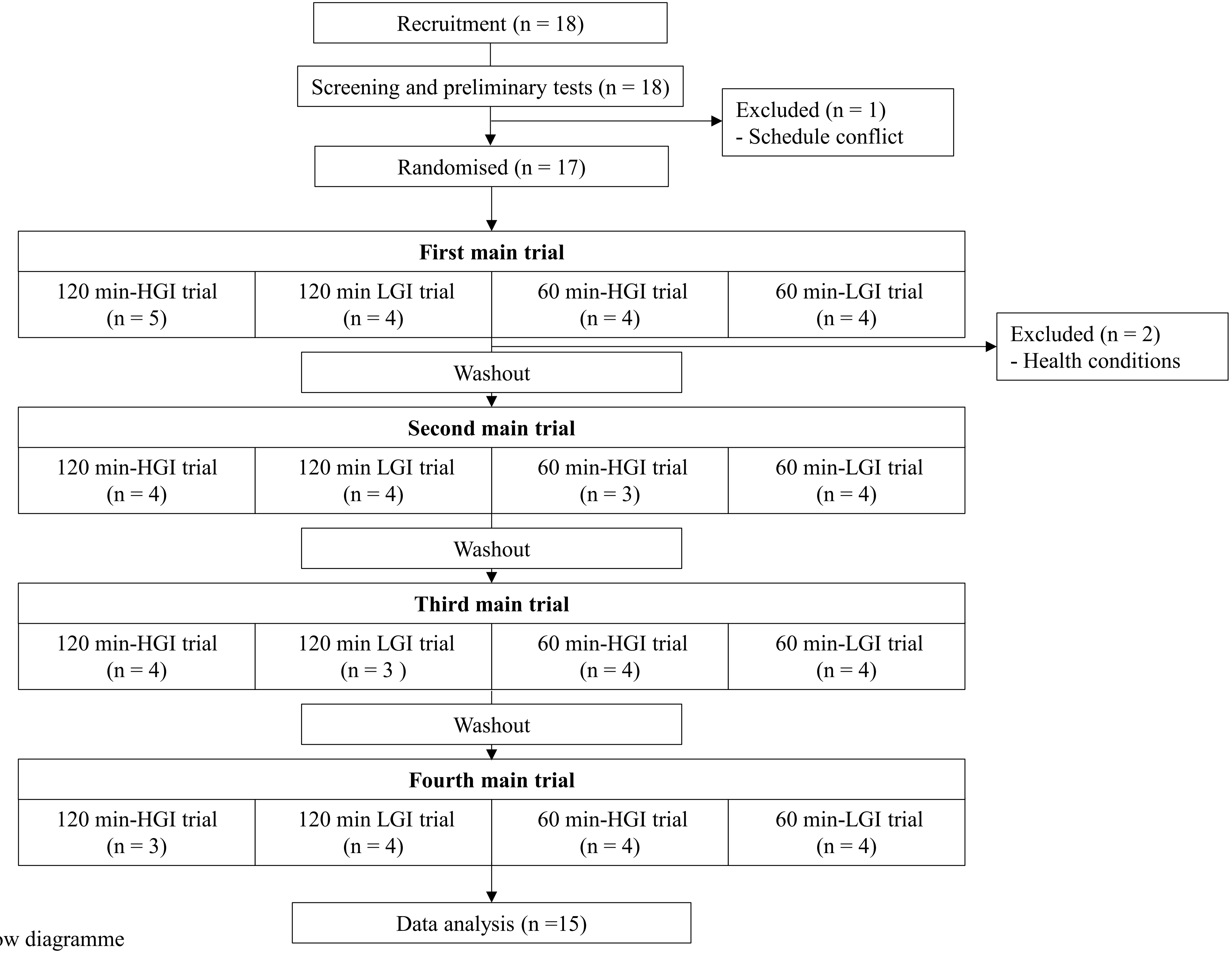

Supplemental Figure 1. A participant flow diagramme
HGI, high glycaemic index; LGI, low glycaemic index.

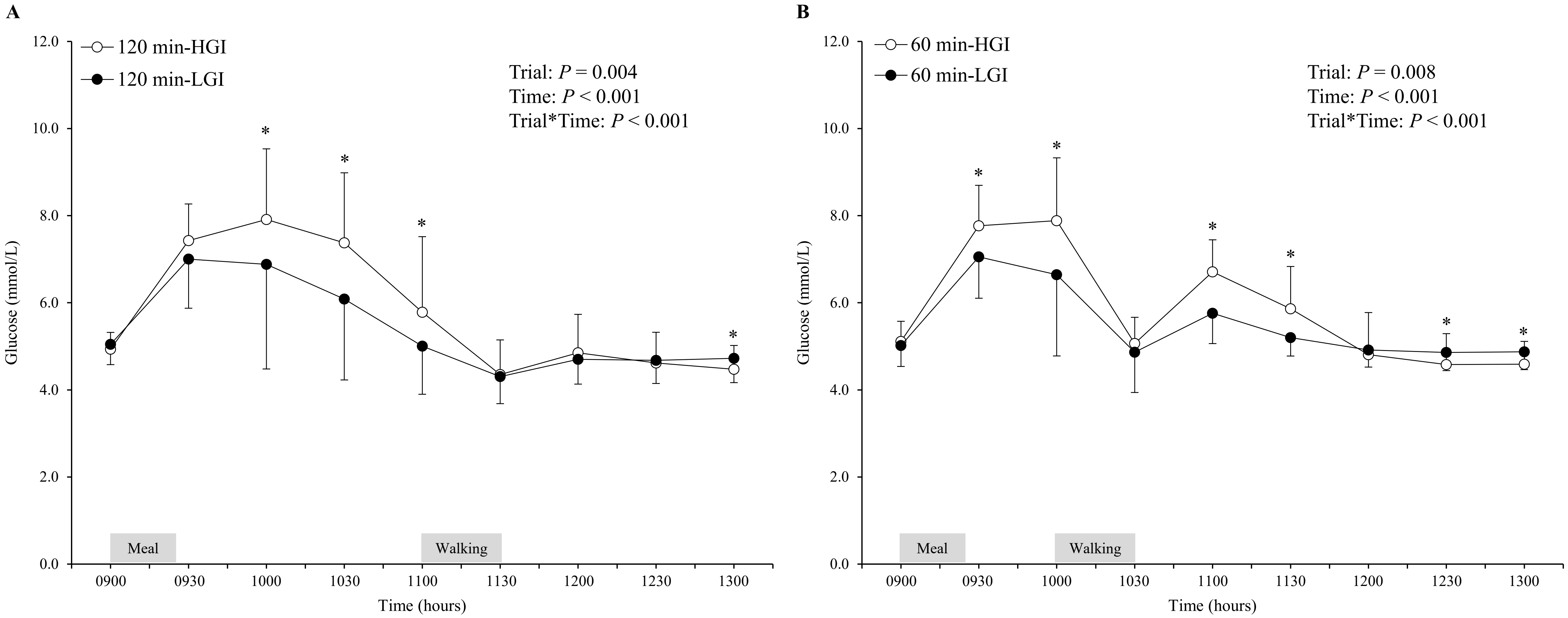

Supplemental Figure 2. Glucose concentrations in the 120 min-HGI and 120 min-LGI trials (A), and the 60 min-HGI and 60 min-LGI trials (B)

Values are means ± standard deviation represented by unidirectional bars. Values were compared using generalised estimating equations. Post-hoc analysis was adjusted for multiple comparisons using the Bonferroni method. *Significantly different between trials at the same time point, $P \leq 0.04$. HGI, high glycaemic index; LGI, low glycaemic index.

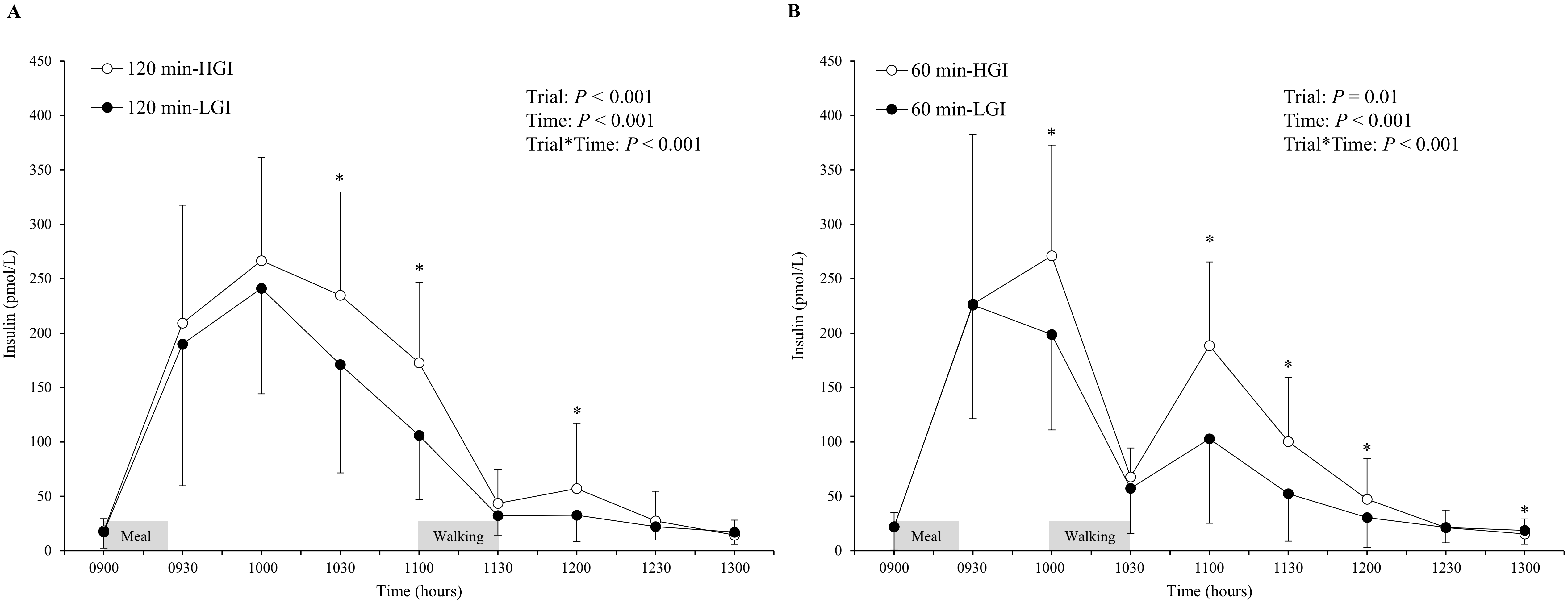

Supplemental Figure 3. Insulin concentrations in the 120 min-HGI and 120 min-LGI trials (A), and the 60 min-HGI and 60 min-LGI trials (B)

Values are means ± standard deviation represented by unidirectional bars. Values were compared using generalised estimating equations. Post-hoc analysis was adjusted for multiple comparisons using the Bonferroni method. *Significantly different between trials at the same time point, $P \leq 0.02$. HGI, high glycaemic index; LGI, low glycaemic index.

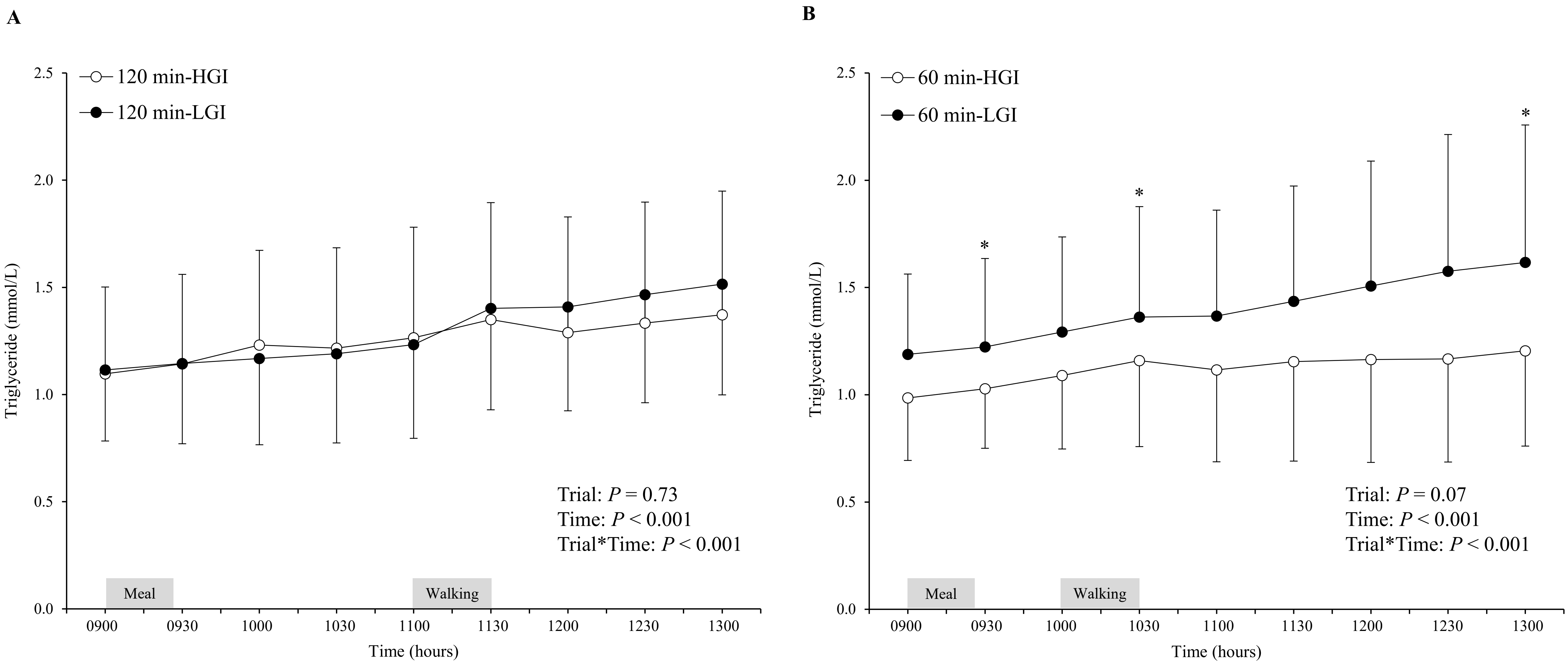

Supplemental Figure 4. Triglyceride concentrations in the 120 min-HGI and 120 min-LGI trials (A), and the 60 min-HGI and 60 min-LGI trials (B)

Values are means ± standard deviation represented by unidirectional bars. Values were compared using generalised estimating equations. Post-hoc analysis was adjusted for multiple comparisons using the Bonferroni method. *Significantly different between trials at the same time point, $P \leq 0.04$. HGI, high glycaemic index; LGI, low glycaemic index.

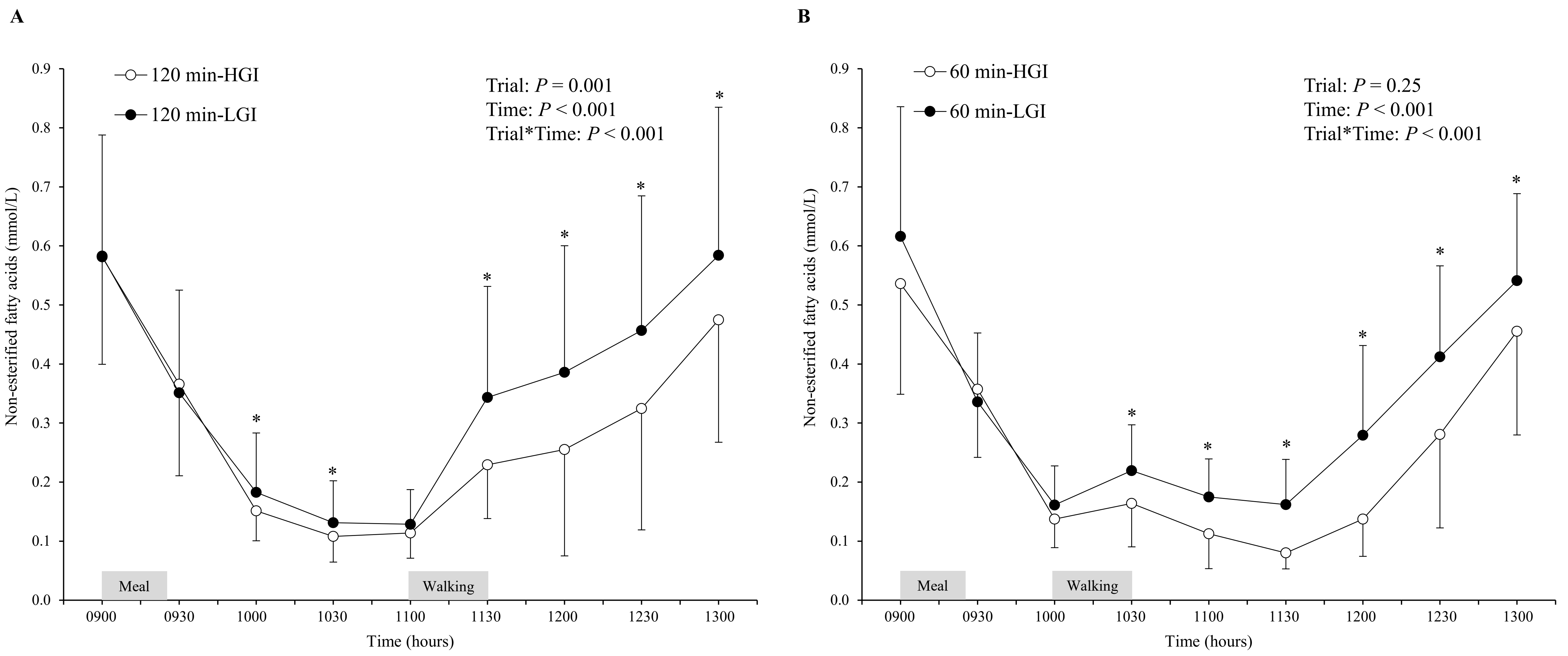

Supplemental Figure 5. Non-esterified fatty acid concentrations in the 120 min-HGI and 120 min-LGI trials (A), and the 60 min-HGI and 60 min-LGI trials (B)

Values are means ± standard deviation represented by unidirectional bars. Values were compared using generalised estimating equations. Post-hoc analysis was adjusted for multiple comparisons using the Bonferroni method. *Significantly different between trials at the same time point, $P \leq 0.04$. HGI, high glycaemic index; LGI, low glycaemic index.

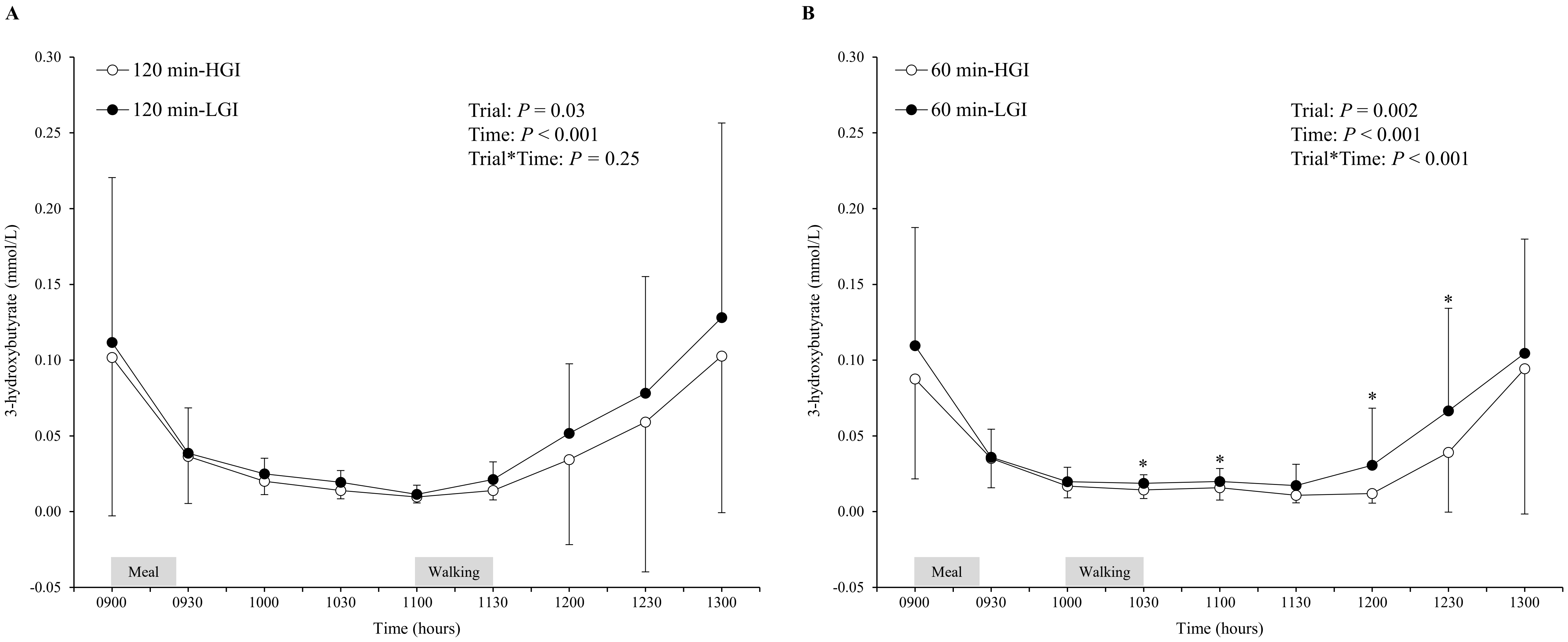

Supplemental Figure 6. 3-hydroxybutyrate concentrations in the 120 min-HGI and 120 min-LGI trials (A), and the 60 min-HGI and 60 min-LGI trials (B)

Values are means ± standard deviation represented by unidirectional bars. Values were compared using generalised estimating equations. Post-hoc analysis was adjusted for multiple comparisons using the Bonferroni method. *Significantly different between trials at the same time point, $P \leq 0.05$. HGI, high glycaemic index; LGI, low glycaemic index.

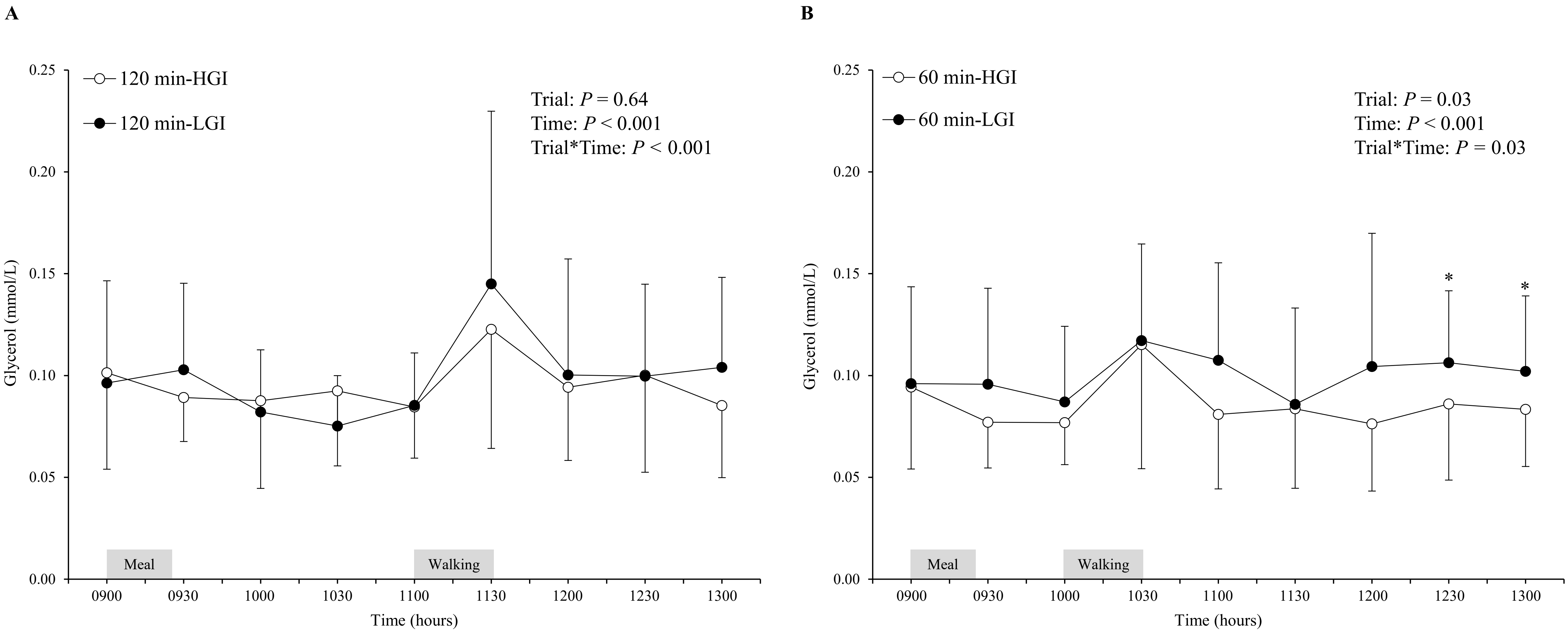

Supplemental Figure 7. Glycerol concentrations in the 120 min-HGI and 120 min-LGI trials (A), and the 60 min-HGI and 60 min-LGI trials (B)

Values are means ± standard deviation represented by unidirectional bars. Values were compared using generalised estimating equations. Post-hoc analysis was adjusted for multiple comparisons using the Bonferroni method. *Significantly different between trials at the same time point, $P \leq 0.02$. HGI, high glycaemic index; LGI, low glycaemic index.

Supplemental Table 1. Fat and carbohydrate oxidation rates in the 120 min-HGI and 120 min-LGI trials (A), and the 60 min-HGI and 60 min-LGI trials (B)

**A**		**Rest**				**Exercise**			**Rest**		
	**Time (hours)**	**0900**	**0930**	**1000**	**1030**	**1110**	**1120**	**1130**	**1200**	**1230**	**1300**
Fat oxidation (g/min)	120 min-HGI	0.09 ± 0.04	0.08 ± 0.04	0.09 ± 0.06	0.08 ± 0.04	0.21 ± 0.08	0.22 ± 0.10	0.23 ± 0.11	0.07 ± 0.03	0.07 ± 0.03	0.08 ± 0.10
	120 min-LGI	0.09 ± 0.04	0.06 ± 0.04	0.07 ± 0.03	0.08 ± 0.03	0.24 ± 0.10	0.27 ± 0.10	0.28 ± 0.09	0.10 ± 0.03*	0.09 ± 0.03	0.10 ± 0.03
Carbohydrate oxidation (g/min)	120 min-HGI	0.05 ± 0.10	0.11 ± 0.12	0.10 ± 0.14	0.11 ± 0.10	0.52 ± 0.34	0.51 ± 0.27	0.47 ± 0.31	0.08 ± 0.07	0.07 ± 0.08	0.08 ± 0.18
	120 min-LGI	0.06 ± 0.06	0.19 ± 0.10*	0.14 ± 0.07	0.11 ± 0.06	0.50 ± 0.31	0.48 ± 0.27	0.42 ± 0.25	0.04 ± 0.05	0.04 ± 0.05	0.02 ± 0.04

**B**		**Rest**		**Exercise**			**Rest**				
	**Time (hours)**	**0900**	**0930**	**1010**	**1020**	**1030**	**1100**	**1130**	**1200**	**1230**	**1300**
Fat oxidation (g/min)	60 min-HGI	0.08 ± 0.03	0.06 ± 0.02	0.21 ± 0.09	0.22 ± 0.09	0.24 ± 0.09	0.08 ± 0.03	0.08 ± 0.03	0.07 ± 0.02	0.09 ± 0.03	0.09 ± 0.03
	60 min-LGI	0.09 ± 0.03	0.05 ± 0.02	0.22 ± 0.09	0.23 ± 0.11	0.24 ± 0.12	0.08 ± 0.03	0.08 ± 0.03	0.08 ± 0.03	0.08 ± 0.04	0.08 ± 0.04
Carbohydrate oxidation (g/min)	60 min-HGI	0.06 ± 0.03	0.11 ± 0.06	0.54 ± 0.21	0.54 ± 0.23	0.53 ± 0.23	0.12 ± 0.08	0.10 ± 0.06	0.09 ± 0.06	0.04 ± 0.06	0.03 ± 0.05
	60 min-LGI	0.06 ± 0.04	0.16 ± 0.08*	0.57 ± 0.22	0.58 ± 0.28	0.57 ± 0.30	0.10 ± 0.07	0.08 ± 0.06	0.07 ± 0.06	0.07 ± 0.06	0.07 ± 0.08

Values are means ± standard deviation. Values were compared using generalised estimating equations and post-hoc analysis was adjusted for multiple comparisons using the Bonferroni method. *Significantly different between trials at the same time point, $P \leq 0.007$ (for 120 min-HGI and 120 min-LGI trials(A)), $P = 0.02$ (for 60 min-HGI and 60 min-LGI trials (B)). HGI, high glycaemic index; LGI, low glycaemic index.

Supplemental Table 2. Respiratory exchange ratios in the 120 min-HGI and 120 min-LGI trials (A), and the 60 min-HGI and 60 min-LGI trials (B)

**A**		**Rest**				**Exercise**			**Rest**		
	**Time (hours)**	**0900**	**0930**	**1000**	**1030**	**1110**	**1120**	**1130**	**1200**	**1230**	**1300**
Respiratory exchange ratio	120 min-HGI	0.76 ± 0.06	0.81 ± 0.06	0.81 ± 0.06	0.82 ± 0.06	0.84 ± 0.05	0.84 ± 0.07	0.84 ± 0.09	0.78 ± 0.08	0.79 ± 0.09	0.74 ± 0.07
	120 min-LGI	0.77 ± 0.07	0.87 ± 0.08*	0.83 ± 0.07	0.81 ± 0.05	0.83 ± 0.06	0.82 ± 0.06	0.81 ± 0.05	0.75 ± 0.05	0.75 ± 0.05	0.73 ± 0.04

**B**		**Rest**		**Exercise**			**Rest**				
	**Time (hours)**	**0900**	**0930**	**1010**	**1020**	**1030**	**1100**	**1130**	**1200**	**1230**	**1300**
Respiratory exchange ratio	60 min-HGI	0.78 ± 0.04	0.82 ± 0.06	0.85 ± 0.06	0.84 ± 0.06	0.84 ± 0.06	0.80 ± 0.05	0.80 ± 0.06	0.79 ± 0.05	0.75 ± 0.06	0.74 ± 0.06
	60 min-LGI	0.77 ± 0.04	0.86 ± 0.05*	0.85 ± 0.06	0.85 ± 0.07	0.84 ± 0.08	0.80 ± 0.06	0.79 ± 0.06	0.78 ± 0.06	0.79 ± 0.08	0.78 ± 0.09

Values are means ± standard deviation. Values were compared using generalised estimating equations and post-hoc analysis was adjusted for multiple comparisons using the Bonferroni method. *Significantly different between trials at the same time point, P = 0.03 (for 120 min-HGI and 120 min-LGI trials(A)), P = 0.02 (for 60 min-HGI and 60 min-LGI trials (B)). HGI, high glycaemic index; LGI, low glycaemic index.

Supplemental Table 3. Subjective appetite in the 120 min-HGI and 120 min-LGI trials (A), and the 60 min-HGI and 60 min-LGI trials (B)

A						Exercise				
	**Time (hours)**	**0900**	**0930**	**1000**	**1030**	**1100**	**1130**	**1200**	**1230**	**1300**
**Satiety (mm)**	120 min-HGI	67.7 ± 30.5	10.1 ± 13.9	16.2 ± 17.5	17.3 ± 17.4	23.2 ± 19.6	33.1 ± 23.3	34.1 ± 24.4	42.3 ± 29.7	49.7 ± 31.0
	120 min-LGI	67.2 ± 25.3	10.6 ± 17.4	15.9 ± 19.8	17.5 ± 16.7	23.1 ± 20.3	30.6 ± 28.9	34.9 ± 28.4	45.7 ± 31.0	53.9 ± 32.6
**Fullness (mm)**	120 min-HGI	24.3 ± 25.7	73.9 ± 26.3	68.2 ± 24.5	66.3 ± 24.9	59.2 ± 25.5	55.1 ± 23.6	49.1 ± 26.7	39.4 ± 25.5	41.3 ± 24.7
	120 min-LGI	20.8 ± 19.9	81.3 ± 20.2	74.2 ± 22.9	75.8 ± 16.1	63.8 ± 24.4	60.1 ± 27.5	55.9 ± 26.8	50.9 ± 28.1	41.3 ± 29.9
**Hunger (mm)**	120 min-HGI	23.7 ± 25.2	83.7 ± 14.9	80.5 ± 15.9	78.7 ± 11.9	70.0 ± 16.8	65.7 ± 17.1	57.7 ± 25.7	43.7 ± 28.1	48.3 ± 26.5
	120 min-LGI	28.3 ± 26.8	85.5 ± 18.4	80.5 ± 15.4	78.2 ± 15.6	75.0 ± 18.8	63.7 ± 23.7	58.0 ± 24.6	48.9 ± 27.3	43.8 ± 29.1
**Prospective food intake (mm)**	120 min-HGI	75.1 ± 19.3	18.1 ± 17.6	23.3 ± 18.8	31.2 ± 25.8	38.7 ± 26.4*	42.3 ± 26.2	45.0 ± 27.3	51.4 ± 29.7	56.0 ± 26.7
	120 min-LGI	73.2 ± 22.6	21.1 ± 25.1	21.5 ± 20.7	24.3 ± 21.5	28.6 ± 20.9	41.5 ± 28.4	43.9 ± 26.7	48.6 ± 30.9	55.9 ± 30.5
**Subjective appetite score**	120 min-HGI	26.3 ± 23.0	82.3 ± 16.0	77.3 ± 16.0	74.1 ± 15.5	66.8 ± 17.2*	61.4 ± 19.9	56.9 ± 24.1	47.4 ± 26.0	46.0 ± 25.9
	120 min-LGI	27.2 ± 20.5	83.8 ± 19.0	79.3 ± 19.0	78.1 ± 16.4	71.8 ± 18.3	62.9 ± 24.8	58.8 ± 24.9	51.4 ± 27.2	43.8 ± 29.7

B				Exercise						
	**Time (hours)**	**0900**	**0930**	**1000**	**1030**	**1100**	**1130**	**1200**	**1230**	**1300**
**Satiety (mm)#**	60 min-HGI	64.1 ± 29.5	12.5 ± 20.4	14.9 ± 19.4	16.5 ± 13.3	20.0 ± 18.4	27.5 ± 22.8	36.6 ± 25.8	50.1 ± 26.9	63.0 ± 20.6
	60 min-LGI	59.1 ± 33.9	13.2 ± 18.2	15.5 ± 17.2	21.7 ± 19.9	24.5 ± 17.1	31.2 ± 18.1	41.1 ± 22.8	52.9 ± 25.6	57.6 ± 24.5
**Fullness (mm)**	60 min-HGI	24.7 ± 25.1	81.1 ± 19.9	78.2 ± 18.3*	76.1 ± 18.5*	72.3 ± 18.9	67.8 ± 17.7*	63.2 ± 21.2	53.1 ± 25.5	51.9 ± 24.3
	60 min-LGI	20.1 ± 19.2	81.7 ± 20.1	70.9 ± 24.6	68.3 ± 24.0	68.7 ± 20.6	56.9 ± 22.3	55.9 ± 24.3	51.4 ± 24.3	41.8 ± 26.7
**Hunger (mm)**	60 min-HGI	24.0 ± 23.8	83.9 ± 16.9	81.9 ± 14.8	80.7 ± 12.0	73.2 ± 19.5	66.9 ± 18.0	59.7 ± 21.7	45.6 ± 23.7	44.1 ± 23.8
	60 min-LGI	16.9 ± 17.3	86.0 ± 15.0	78.9 ± 16.8	72.3 ± 18.5	69.9 ± 18.2	62.7 ± 22.0	58.7 ± 20.4	52.2 ± 24.1	42.1 ± 23.1
**Prospective food intake (mm)**	60 min-HGI	74.3 ± 22.7	18.2 ± 17.4	21.9 ± 18.4	18.4 ± 19.3	29.8 ± 25.6	38.0 ± 24.7	44.6 ± 24.4	53.6 ± 24.9	57.7 ± 23.9
	60 min-LGI	77.4 ± 15.0	20.2 ± 20.9	18.1 ± 16.9	28.3 ± 20.2	30.1 ± 20.6	35.2 ± 19.3	44.9 ± 22.2	51.2 ± 22.6	55.6 ± 24.2
**Subjective appetite score**	60 min-HGI	27.6 ± 23.6	83.6 ± 17.8	80.8 ± 15.6	80.5 ± 13.6	73.9 ± 18.2	67.3 ± 17.7	60.4 ± 19.9	48.8 ± 20.1	43.8 ± 18.7
	60 min-LGI	25.1 ± 17.4	83.6 ± 17.1	79.1 ± 17.3	72.6 ± 18.7	71.0 ± 17.9	63.3 ± 18.1	57.2 ± 20.8	49.9 ± 21.0	42.7 ± 22.8

Values are means ± standard deviation. Values were compared using generalised estimating equations and post-hoc analysis was adjusted for multiple comparisons using the Bonferroni method. #Significantly different between trials, a main effect of trial, $P = 0.01$. *Significantly different between trials at the same time point, $P \leq 0.05$ (for 120 min-HGI and 120 min-LGI trials (A)), $P \leq 0.04$ (for 60 min-HGI and 60 min-LGI trials (B)). HGI, high glycaemic index; LGI, low glycaemic index.
